# Quantitative NMR for detection of spinosad residues in agricultural soils†

**DOI:** 10.1039/d5ra00356c

**Published:** 2025-02-18

**Authors:** Tushar Janardan Pawar, Siuly Xenia Ramos-Cruz, Israel Bonilla-Landa, Ghazala Muteeb, Enrique Delgado-Alvarado, Sachin V. Patil, Irving David Perez-Landa, José Luis Olivares-Romero

**Affiliations:** a Red de Estudios Moleculares Avanzados, Instituto de Ecología A.C., Carretera Antigua a Coatepec 351 Xalapa Veracruz 91073 Mexico jose.olivares@inecol.mx; b Tecnológico Nacional de México/Instituto Tecnológico de Boca del Río Carretera Veracruz-Córdoba 12 Boca del Río Veracruz 94290 Mexico; c Department of Nursing, College of Applied Medical Sciences, King Faisal University Al-Ahsa Saudi Arabia; d Micro and Nanotechnology Research Center, Universidad Veracruzana Blvd. Av. Ruiz Cortines No. 455 Fracc. Costa Verde Boca del Río Veracruz 94294 Mexico; e Department of Chemistry, Research Centre HPT Arts and RYK Science College (Affiliated to S. P. Pune University) Nashik Maharashtra 422005 India

## Abstract

Monitoring pesticide residues in soil is crucial for ensuring food safety and environmental sustainability. Spinosad, widely used in sustainable agriculture due to its selective toxicity and reduced environmental impact, poses detection challenges with traditional chromatographic methods, which require extensive sample preparation and are destructive. This study evaluates quantitative nuclear magnetic resonance (qNMR) as a non-destructive, efficient method for spinosad quantification in soil samples, emphasizing its potential for routine environmental monitoring. The qNMR method was validated with an 88% recovery rate for spinosad in agricultural soils, a limit of detection (LOD) of 0.0414 mg mL^−1^, and a limit of quantification (LOQ) of 0.1254 mg mL^−1^. The method exhibited linearity across a 2–8 mg mL^−1^ concentration range (*R*^2^ = 0.9928) and high precision, with coefficients of variation below 1% for both intraday and interday analyses. It was adaptable to diverse soil types, achieving consistent quantification in red loamy soil from Veracruz and black organic soil from Querétaro, Mexico. These results establish qNMR as a reliable, cost-effective alternative to chromatographic methods for spinosad residue analysis in soil, supporting routine environmental monitoring and regulatory compliance in sustainable agriculture.

## Abstract 2

Monitoring pesticide residues in soil is crucial for ensuring food safety and environmental sustainability.

## Graphical abstract

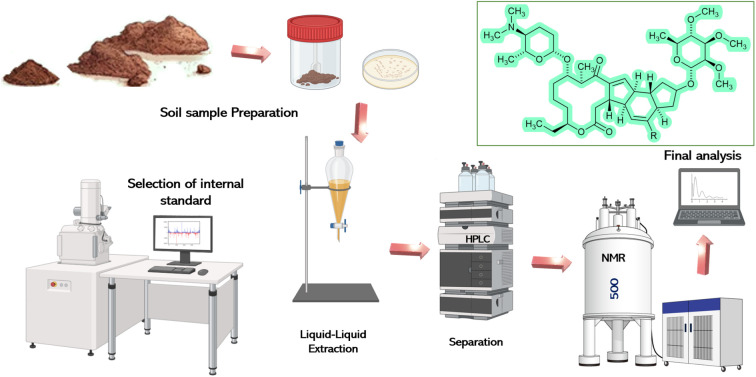

## Introduction

Ensuring the safety of agricultural products necessitates reliable detection of pesticide residues, including bioinsecticides like spinosad, which, despite their environmentally friendly profile, can still accumulate in soil. Regulatory agencies set Maximum Residue Limits (MRLs) based on safety assessments and good agricultural practices to prevent excessive exposure to pesticide residues. However, MRLs are not direct measures of human health impact; rather, they act as thresholds for compliance in agricultural practices.^1^ Spinosad, a naturally derived bioinsecticide effective against various insect pests, has gained popularity due to its selective toxicity and reduced environmental impact compared to synthetic pesticides.^2^ However, even bioinsecticides can leave residues in agricultural soils, which may persist in the environment and eventually accumulate in food products, posing risks to food safety and environmental sustainability.^3^ Monitoring pesticide residues, including bioinsecticides, in agricultural soils is therefore essential for maintaining the safety of the food supply and ensuring compliance with regulatory standards that govern residue limits.^4^

**Fig. 1:**
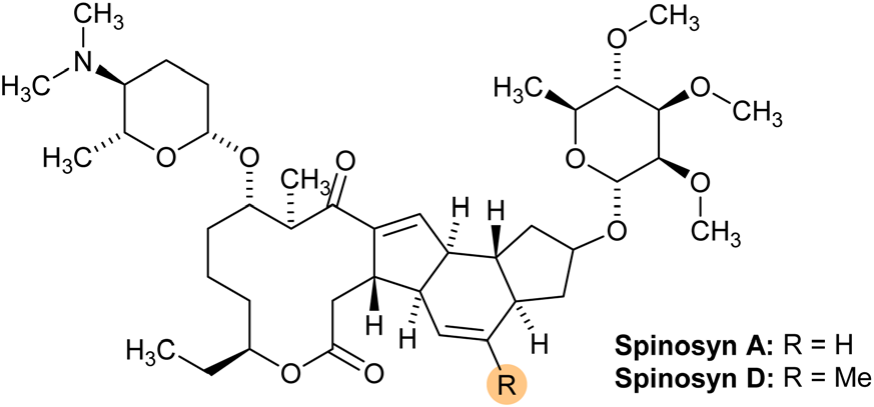
Structure of spinosad, highlighting spinosyn A (R = H) and spinosyn D (R = Me).

Spinosad is a naturally derived bioinsecticide produced by the fermentation of *Saccharopolyspora spinosa*, a soil-dwelling actinobacterium.^5^ It consists primarily of two active ingredients: spinosyn A and spinosyn D (Fig. 1).^6^ Spinosad has been widely adopted for controlling insect pests in various crops due to its low toxicity to mammals and its effectiveness against a broad range of insect species, including those that have developed resistance to conventional chemical pesticides.^2^ Despite these advantages, there are concerns about the persistence of spinosad residues in soils and the potential for these residues to enter the food chain through contamination of crops. Therefore, it is essential to have reliable methods for detecting and quantifying spinosad residues in agricultural soils to ensure that residue levels remain within safe limits and to mitigate any potential risks to public health and the environment.^7^

Traditional analytical methods for pesticide residue detection, such as gas chromatography (GC) and high-performance liquid chromatography (HPLC), have long been used to monitor pesticide residues in food and environmental samples.^8^ While these techniques offer high sensitivity and accuracy, they often require extensive sample preparation, derivatization, and solvent use, which can be time-consuming and costly.^9^ Moreover, these techniques are destructive, meaning the samples cannot be recovered after analysis. In the context of large-scale agricultural monitoring, there is a need for faster, more efficient methods that minimize sample preparation and allow for non-destructive analysis.

Quantitative nuclear magnetic resonance (qNMR) spectroscopy has emerged as a powerful alternative for the detection and quantification of chemicals in complex matrices.^10^ Unlike chromatography-based methods, qNMR does not require extensive sample preparation, is non-destructive, and provides direct quantification of analytes without the need for external calibration standards.^11^ These characteristics make qNMR particularly attractive for routine monitoring of pesticide residues in agricultural soils. qNMR relies on the proportionality between the integrated areas of NMR signals and the number of nuclei in the sample, allowing for accurate quantification of analytes over a wide concentration range.^10,12^

In recent years, qNMR has been increasingly used in various fields, including pharmaceuticals, food safety, and environmental monitoring.^13^ Its application to pesticide residue analysis, however, is still relatively limited. A few studies have demonstrated the utility of qNMR for detecting pesticide residues in water, food, and biological samples,^4,13^ but its use in soil samples, particularly for bioinsecticides like spinosad, remains underexplored. Given the advantages of qNMR, it holds great potential for improving the efficiency and accuracy of pesticide residue monitoring in agricultural soils.

The ability to monitor pesticide residues in soil is not only important for environmental safety but also for ensuring that food production practices meet regulatory standards.^14^ In many countries, maximum residue limits (MRLs) for pesticides are established to protect consumers from potential health risks associated with pesticide exposure.^15^ Exceeding these limits can lead to the rejection of agricultural products in both domestic and international markets, resulting in significant economic losses for farmers and exporters. Furthermore, monitoring soil residues is critical for understanding the persistence of pesticides in the environment and their potential long-term effects on soil health and biodiversity.

The European Union (EU), for example, has established strict regulations on the use of pesticides and bioinsecticides in agriculture, requiring regular monitoring of residue levels in soil, water, and food products. Similarly, the U.S. Environmental Protection Agency (EPA) mandates the monitoring of pesticide residues to ensure compliance with safety standards.^16^ In this context, the development of robust and efficient analytical methods, such as qNMR, is essential for supporting regulatory compliance and promoting sustainable agricultural practices.

This study aims to evaluate the applicability of qNMR for detecting and quantifying spinosad residues in agricultural soils by employing both 1D and 2D NMR techniques. We seek to demonstrate that qNMR can provide accurate and reproducible results with minimal sample preparation, making it an ideal tool for routine monitoring in agricultural settings. The use of qNMR offers significant advantages in terms of cost-effectiveness, as it eliminates the need for expensive chemical reagents and calibration standards typically required for chromatography-based methods. Additionally, qNMR's non-destructive nature allows for the preservation of samples, which is particularly beneficial for environmental monitoring, where the same sample may need to undergo multiple analyses for various contaminants or for long-term studies assessing the impact of pesticide use on soil health. In this study, we applied qNMR to the detection of spinosad residues in soil samples collected from agricultural fields, evaluating the method's accuracy, precision, and linearity across a range of spinosad concentrations, with a focus on its applicability for routine monitoring. The study demonstrates that qNMR is a reliable and efficient method for pesticide residue analysis, with the potential to be widely adopted for environmental and food safety testing in agriculture.

## Results and discussion

### Purification of spinosad

Spinosad was successfully purified through a multi-step process involving liquid–liquid extraction from two commercial products, followed by column chromatography and Thin Layer Chromatography (TLC) for compound identification. The TLC analysis revealed the presence of spinosad under UV light at 254 nm, confirmed by the characteristic blue coloration upon staining with ammonium molybdate. The retention factor (*R*_f_) of the purified spinosad was measured at 0.4, consistent with previous reports.^11^

Notable differences were observed between the spinosad obtained from Spintor 12SC® and Gadeon 12SC®. The product purified from Spintor 12SC® resulted in a fine, white crystalline powder, while the Gadeon 12SC® product yielded a denser, yellowish material, suggesting potential variations in formulation, purity, or the presence of impurities. The final masses obtained were 1.82 g for Spintor 12SC® and 1.162 g for Gadeon 12SC®, both of which were lower than the expected theoretical values, potentially due to extraction inefficiencies or residual impurities.

### Separation of spinosyns

**Fig. 2:**
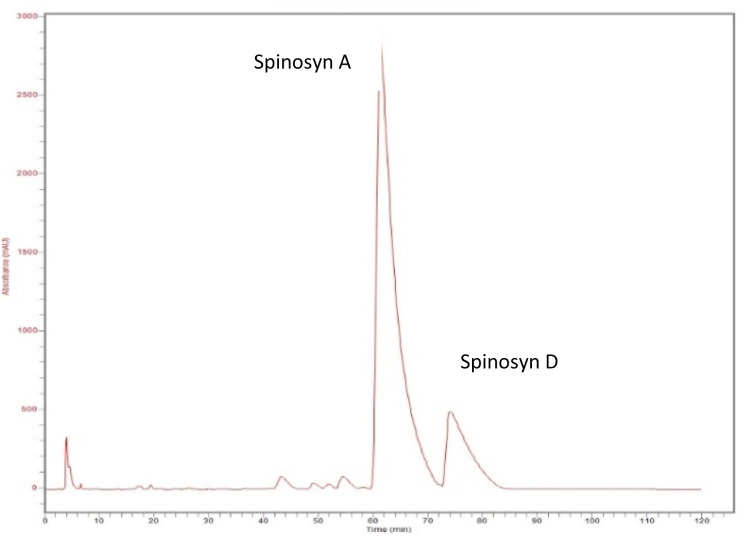
HPLC chromatogram showing the separation of spinosyns A (retention time: 62 minutes) and D (retention time: 76 minutes).

Spinosyns A and D were successfully separated using a reverse-phase HPLC technique. The separation was achieved using a C18 reverse-phase column with a mobile phase of acetonitrile and water (70 : 30 v/v) at a flow rate of 1.0 mL min^−1^, with UV detection at 254 nm. The distinct retention times of 62 minutes for spinosyn A and 76 minutes for spinosyn D (Fig. 2) allowed for clear identification of both compounds. These results closely align with previously reported retention times by Pérez-Landa *et al.*, 2021,^11^ and DeAmicis *et al.*, 2017,^17^ confirming the stability of spinosad during sample handling and analysis. Fig. 2 displays the HPLC chromatogram, illustrating the clear separation of spinosyn A and D.

### NMR spectroscopy analysis

**Fig. 3:**
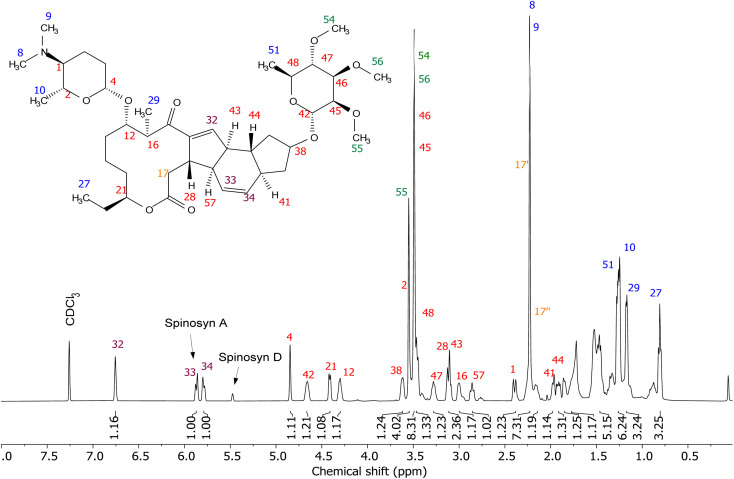
^1^H NMR spectra of spinosad obtained from Spintor 12SC®. The spectra show well-resolved signals for spinosyn A (doublet at 5.87 ppm) and spinosyn D (singlet at 5.47 ppm), confirming the presence of both components.

Spinosad extracted from Spintor 12SC® exhibited distinct NMR signals for spinosyns A and D. Spinosyn A was identified by a doublet at 5.87 ppm, while spinosyn D presented a singlet at 5.47 ppm, demonstrating the precision of the NMR method in distinguishing between these two components (Fig. 3). The choice to focus on spinosad obtained from Spintor 12SC® for further analysis was driven by the clearer definition of spectral peaks and the simpler extraction process, which facilitated higher purity. This approach followed the methodology described by Pérez-Landa *et al.*, 2021,^11^ ensuring the reliability and reproducibility of the NMR spectroscopic analysis.

The 1D ^1^H NMR spectra were recorded using a 500 MHz spectrometer, revealing well-resolved peaks corresponding to the expected chemical shifts of spinosyn A and D, confirming their presence in the extracted samples (Fig. 3). A relaxation delay of 1 second between scans was chosen based on preliminary T1 measurements and multiple trials, ensuring adequate relaxation for accurate signal intensity measurements.

**Fig. 4:**
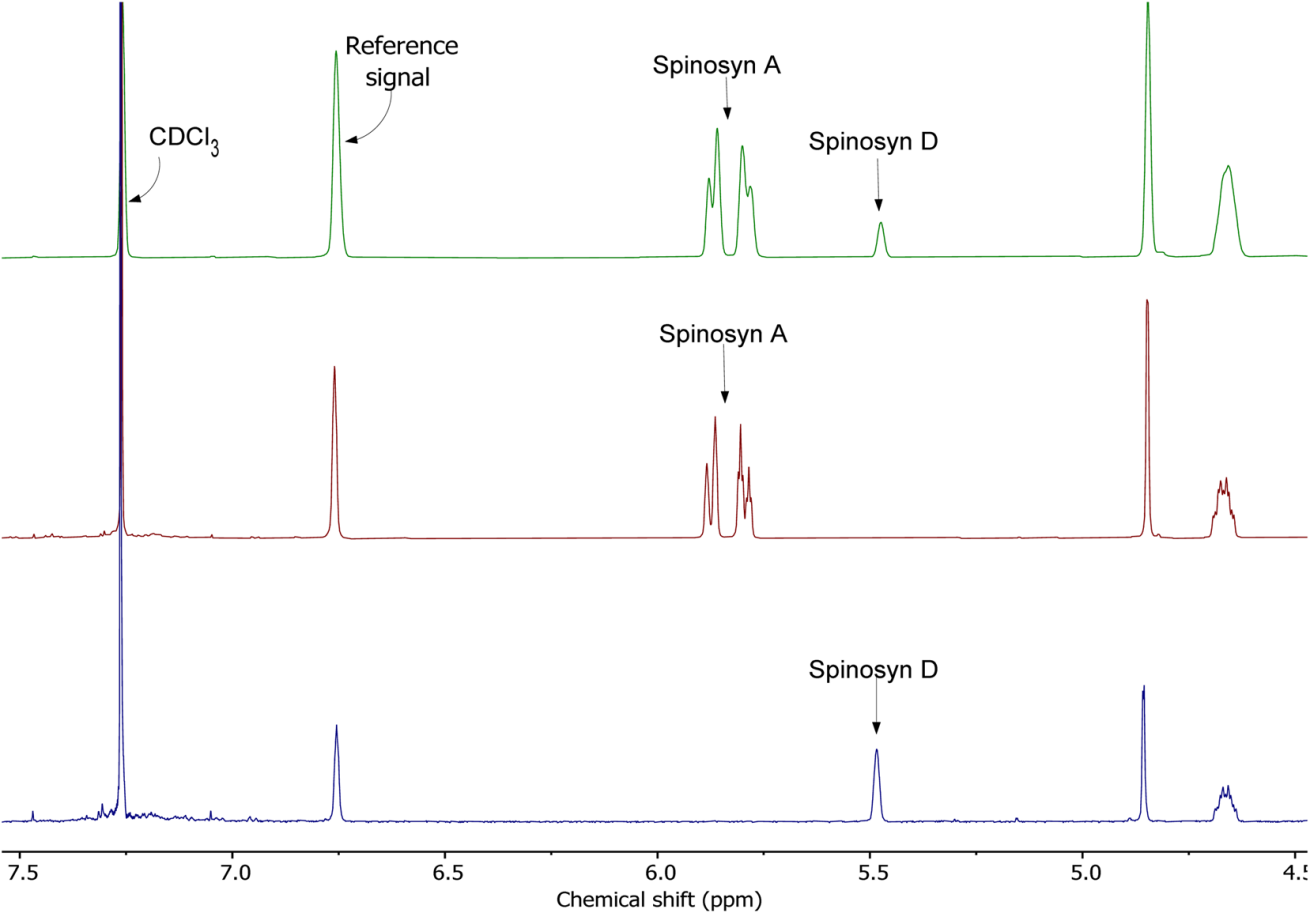
Combined ^1^H NMR spectra of spinosad. The first spectrum (green) represents the mixture of spinosyns, the second spectrum (red) shows spinosyn A, and the third spectrum (blue) displays spinosyn D. Key signals for spinosyn A and spinosyn D are highlighted, along with the reference signal and residual solvent peaks (CDCl_3_).

Fig. 4 presents the combined ^1^H NMR spectra of spinosad. The first spectrum (green) represents the mixture of spinosyns, the second spectrum (red) corresponds to spinosyn A, and the third spectrum (blue) displays spinosyn D. Key signals for each spinosyn are annotated, including the reference signal and residual solvent peaks (CDCl_3_).

The ^13^C NMR data, acquired at 125.70 MHz, provided additional confirmation of the carbon framework in both spinosyns. Although the relatively long acquisition time (5000 scans) was required due to the low natural abundance of ^13^C, the resulting spectra exhibited clear signals for all carbons, further supporting the structural assignments made from the proton data (Page S19, ESI†).

**Fig. 5:**
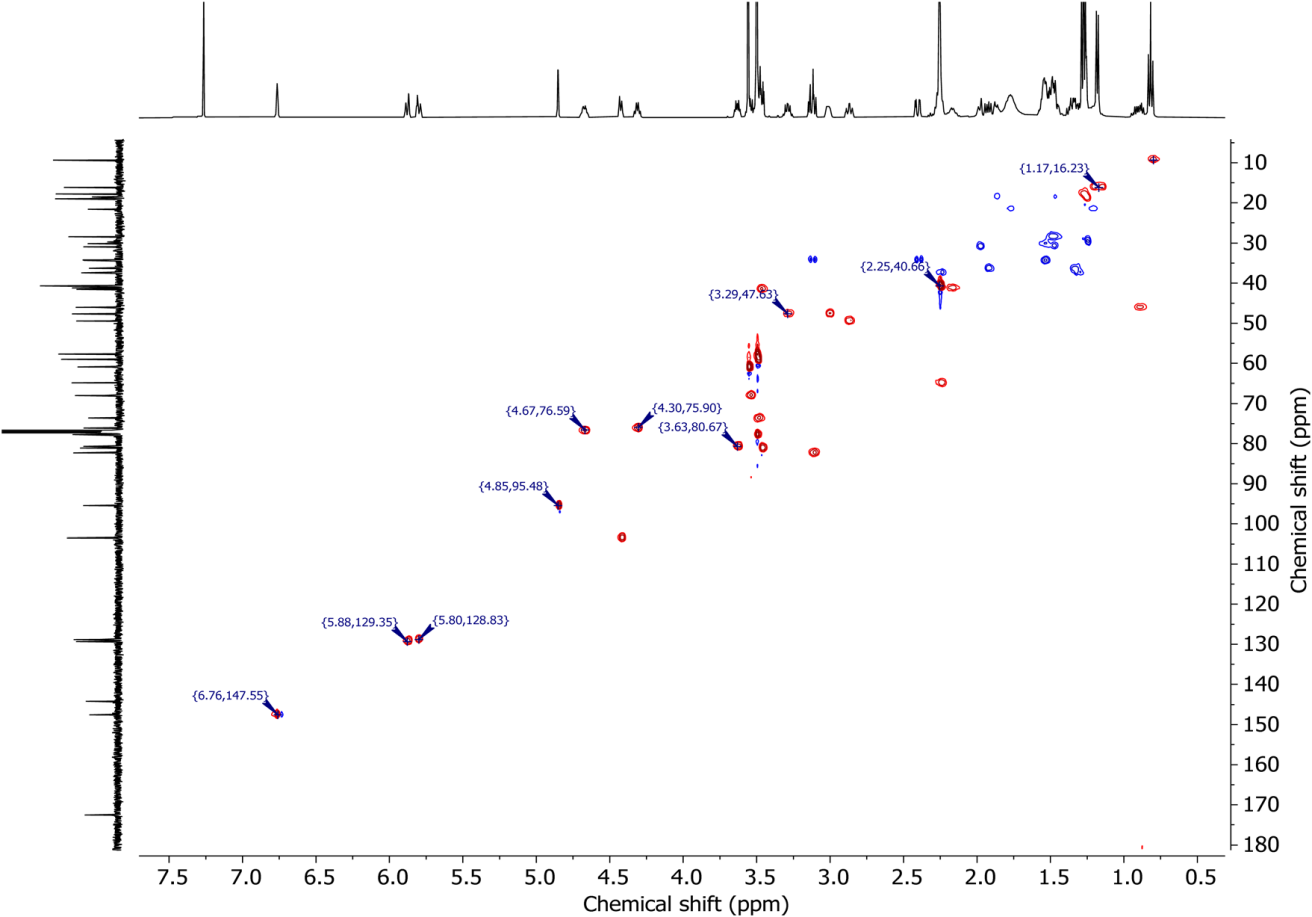
^1^H–^13^C HSQC NMR spectra of spinosyn A showing the correlations between hydrogen and carbon atoms.

To further elucidate the structural relationships within the spinosad components, 2D NMR experiments were employed. COSY spectra (Page S20, ESI†) provided correlations between coupled protons, while ^1^H–^13^C HSQC (Fig. 5) and ^1^H–^13^C HMBC spectra (Page S22, ESI†) offered comprehensive information on proton–carbon couplings and long-range heteronuclear correlations, respectively. The choice of the number of scans and spectral widths allowed for a high signal-to-noise ratio and resolution, providing clear structural connectivity within the molecule. This comprehensive NMR analysis confirmed the identities of both spinosyn A and D, as well as their relative purity in the samples (additional spectra, including for spinosyn A, spinosyn D, the internal standard, and soil samples, are provided in the ESI†).

### Selection of internal standard

**Fig. 6:**
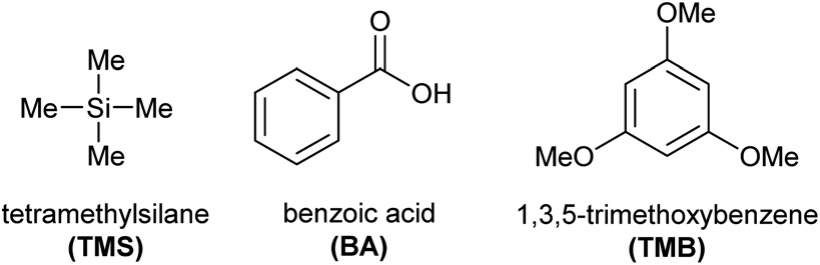
Structures and IUPAC names of compounds used as internal standards: tetramethylsilane (TMS), benzoic acid (BA), and 1,3,5-trimethoxybenzene (TMB). TMB was selected for its non-interference with spinosad signals and its better performance in quantification accuracy.

In selecting an appropriate internal standard for the quantification of spinosad, several candidates were evaluated, including tetramethylsilane (TMS), benzoic acid (BA), and 1,3,5-trimethoxybenzene (TMB) (Fig. 6). Each compound was tested for compatibility with the NMR analysis, particularly ensuring no signal overlap with the key ^1^H and ^13^C NMR signals of spinosyn A and D.

TMB was ultimately selected as the internal standard due to its favorable characteristics, including its distinct chemical shifts, which did not interfere with the signals of spinosad, and its high solubility in CDCl_3_, the solvent used in the study. In addition, TMB provided nearly 97–98% quantification accuracy in preliminary tests, which was superior to the other candidates. This level of accuracy confirmed that TMB is highly suitable for precise and accurate spinosad quantification in both 1D and 2D NMR experiments.

### Quantification of spinosad

The validation of the qNMR method confirmed its effectiveness for quantifying spinosad in soil samples, achieving a recovery rate of 88%. This recovery rate is adequate for detecting and accurately quantifying spinosad residues at relevant concentration levels, supporting the method's applicability for routine environmental monitoring. The high recovery rate ensures that the method meets agricultural standards for bioinsecticide residue analysis, providing a reliable tool for compliance with regulatory guidelines. Furthermore, the qNMR approach offers the advantages of minimal sample preparation and non-destructive analysis, making it suitable for widespread use in sustainable agricultural practices and environmental assessments.

### Method validation

The method underwent rigorous validation, including specificity, linearity, accuracy, precision, detection and quantification limits, and sample stability assessment.^18^ The established parameters and calibration curves underscored the method's robustness, reliability, and applicability for soil sample analysis.

**Fig. 7:**
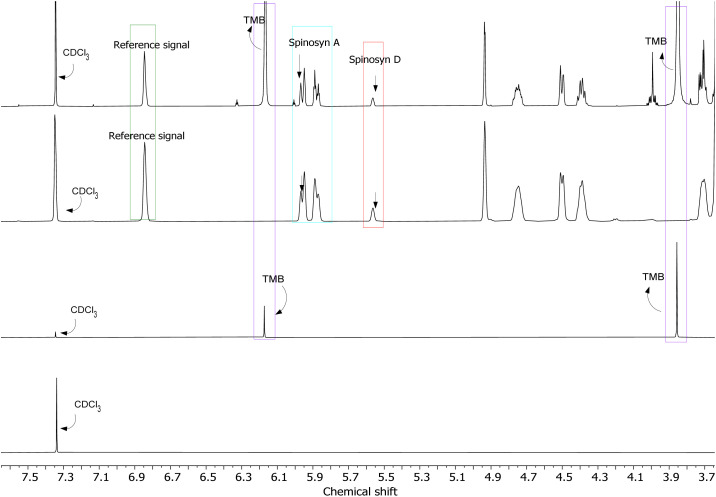
^1^H NMR spectra showing the signals for the reference (CDCl_3_), internal standard (TMB), spinosyn A, and spinosyn D, demonstrating the specificity of the method.

The specificity of the qNMR method was assessed by examining potential interferences between the solvent (CDCl_3_), the internal standard (TMB), and the analyte (Spinosad), as well as any cross-signals within the reference mixture samples (Fig. 7).^19^ The analysis confirmed that there were no significant cross-signals or interferences, ensuring the method's capability to accurately identify spinosad in complex matrices. Additionally, to verify that other soil components would not interfere with spinosad detection, soil samples were tested with different solvents (ethyl acetate, chloroform, and ethanol). No interfering signals were detected in these solvents, confirming that the method could accurately distinguish spinosad in soil matrices (see ESI†).

**Fig. 8:**
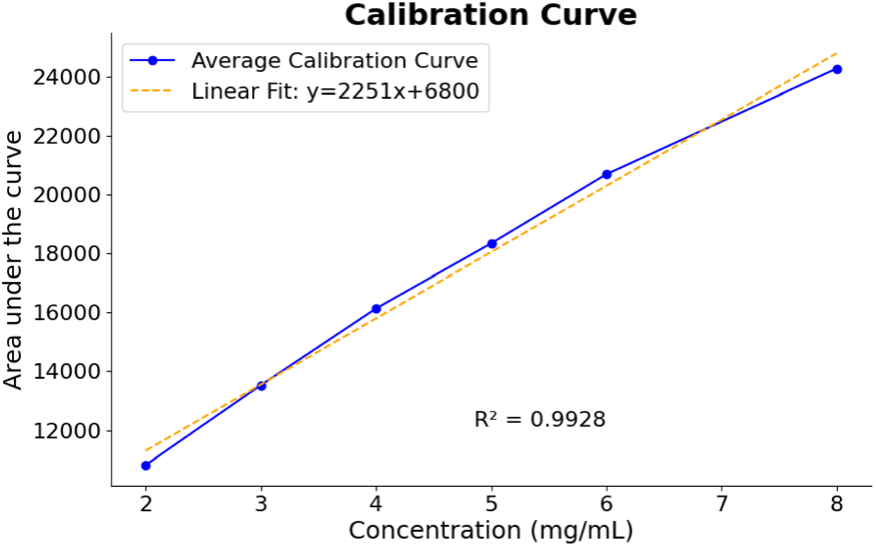
Calibration curve of spinosad showing the average calibration data points and the linear fit (*y* = 2251*x* + 6800) with a regression coefficient (*R*^2^) of 0.9928, indicating strong linearity.

Linearity was demonstrated by constructing a calibration curve based on serial dilutions of a 10 mg mL^−1^ spinosad solution in CDCl_3_/TMB. The equation derived from the calibration curve, *y* = 2251*x* + 6800, represents the relationship between the NMR signal intensity (*y* axis, in arbitrary units) and the spinosad concentration (*x* axis, in mg mL^−1^). This calibration curve exhibited excellent linearity across the concentration range tested (2–8 mg mL^−1^), with a regression coefficient (*R*^2^) exceeding 0.9928, indicating a strong linear correlation between spinosad concentration and NMR signal intensity (Fig. 8).^19,20^ The standard error of the estimate was calculated as 463.841, confirming the precision of the model (see ESI† for residual plot and calculations).

The accuracy and precision of the qNMR method were evaluated using both intraday and interday analyses at concentrations of 3 mg mL^−1^ and 6 mg mL^−1^. Accuracy tests, based on comparing known and measured concentrations, showed an error margin within ±2%, underscoring the method's high accuracy in quantifying spinosad. At 3 mg mL^−1^, the mean error was −0.56%, and at 6 mg mL^−1^, the mean error was 2.76%, demonstrating consistent accuracy across different concentration levels.^21^

Precision was also confirmed by intraday and interday tests. The mean concentrations for intraday analyses at 3 mg mL^−1^ and 6 mg mL^−1^ were 2.983 mg mL^−1^ and 6.155 mg mL^−1^, respectively. The standard deviations were 0.022 mg mL^−1^ for 3 mg mL^−1^ and 0.039 mg mL^−1^ for 6 mg mL^−1^, yielding coefficients of variation (CV) of 0.74% and 0.63%, respectively.^21*a*^ These low CV values highlight the reproducibility and robustness of the qNMR method (see ESI† for detailed accuracy and precision plots).

The Limit of Detection (LOD) and Limit of Quantification (LOQ) were determined using serial dilutions of a 6 mg mL^−1^ stock solution of spinosad. The LOD was calculated at 0.0414 mg mL^−1^, and the LOQ at 0.1254 mg mL^−1^, based on the standard deviation of the response and the slope of the calibration curve.^19^ These results demonstrate the method's sensitivity for detecting and quantifying low concentrations of spinosad in environmental samples, making it suitable for routine monitoring of bioinsecticide residues (see ESI† for detailed calculations).

The stability of the spinosad solution was assessed over a period of 28 hours, with measurements taken at 0, 4, 8, 24, and 28 hours. The concentration of spinosad remained consistent at 0.5 mg mL^−1^ throughout the testing period, showing no significant degradation. Stability tests were performed under controlled conditions (protected from light and at constant temperature), confirming the reliability of the method for long-term sample analysis.^22^

### Laboratory soil sample quantification tests

The quantification of spinosad in soil samples followed a detailed preparation process, beginning with drying, pulverizing, and sieving the soil to ensure uniform bioinsecticide impregnation and to facilitate the extraction process. This preparation was crucial for achieving consistent and reliable NMR analysis results. To maximize the recovery of spinosad, the extraction was performed three times using a carefully selected solvent, followed by filtration to remove any remaining soil particulates. This ensured that the NMR analysis was conducted exclusively on solubilized spinosad, free from interference by soil debris, allowing for accurate quantification.^23^

The study evaluated two distinct soil types to assess the method's adaptability and effectiveness across different agricultural environments. This comparison allowed for the selection of the soil type that provided the most reliable NMR spectral results, highlighting the method's versatility in analyzing soils with varying compositions.^24^ The choice of solvent played a critical role in the extraction and quantification process. Several solvents were tested for their ability to extract spinosad efficiently and for their compatibility with NMR analysis. The selected solvent did not interfere with the NMR signals of spinosad, enabling accurate quantification and underscoring the importance of solvent selection in bioinsecticide residue analysis.

Following the extraction, ^1^H NMR spectroscopy was employed to quantify the recovered spinosad from the soil samples. Specific mathematical equations were used to calculate the concentration and total mass of spinosad present in the soil, thereby validating the efficiency of the extraction and purification processes. The results demonstrated a recovery rate of 88%, confirming the effectiveness of the qNMR method for quantifying spinosad in soil samples. This validation underscores the method's reliability in environmental analysis.

## Discussion

The results of this study underscore the potential of qNMR spectroscopy as a robust and efficient method for quantifying bioinsecticides in soil samples. The successful application of qNMR to quantify spinosad, achieving a recovery rate of approximately 88%, has several important implications. First, the ability to accurately quantify spinosad in soil supports environmental monitoring and regulatory compliance, ensuring that agricultural practices remain sustainable and environmentally responsible. By providing a reliable method for detecting bioinsecticide residues, this study contributes to the development of safer agricultural practices. Additionally, the validated qNMR method sets a foundation for the development of similar techniques for other bioinsecticides and agricultural chemicals, potentially leading to broader applications in environmental chemistry and toxicology. The method's adaptability to various soil types further highlights its potential for monitoring bioinsecticides across different agricultural environments.

One of the key advantages of qNMR is its non-destructive nature, allowing for the preservation of samples for further analysis. This feature is particularly beneficial in environmental studies, where the integrity of the sample is crucial for conducting multiple analyses. Furthermore, qNMR is a relatively rapid and simple technique compared to more complex methods like HPLC or GC-MS, making it an attractive option for routine analysis in both research and regulatory laboratories. The ability to use qNMR without extensive sample preparation or chemical derivatization offers significant advantages over traditional chromatographic methods, such as those used by Pérez-Landa *et al.* (2021)^11^ and DeAmicis *et al.* (2017),^17^ who employed HPLC for spinosyn separation.

The detection and quantification limits (LOD of 0.0414 mg mL^−1^ and LOQ of 0.1254 mg mL^−1^) achieved in this study are competitive with those reported for HPLC and GC-MS, underscoring the sensitivity of the qNMR method. Furthermore, the accuracy, with an error margin of ±2%, and precision, reflected by low standard deviations in both intraday and interday analyses, demonstrate the method's reliability and reproducibility. These results are on par with or surpass those reported for other analytical techniques. While chromatographic methods may provide superior sensitivity for detecting trace levels of spinosad in complex matrices, qNMR offers a direct, non-destructive quantification approach with significant advantages in cost and workflow efficiency, making it particularly suitable for routine monitoring applications.

Despite these advantages, the widespread application of qNMR in agricultural and environmental monitoring is limited by instrument accessibility and the expertise required for spectral interpretation. High-field NMR spectrometers are primarily available in well-equipped laboratories, which may restrict the direct implementation of this technique in routine agricultural settings. To address this limitation, several strategies can be considered, such as establishing centralized analysis centers, developing portable sample preparation kits, and fostering collaborations with universities and research institutions to expand access to qNMR technology. Furthermore, recent advancements in benchtop NMR technology and automated data processing algorithms are improving the accessibility of qNMR for broader applications, including environmental and agricultural analysis. Studies have demonstrated the integration of quantum mechanical calculations with machine learning-based automated analysis, which facilitates qNMR adoption beyond highly specialized laboratories.^25^ These developments suggest that future improvements in instrument accessibility and automation could expand the applicability of qNMR for routine pesticide residue monitoring.

Finally, the results of this study align with the growing advocacy for sustainable agricultural practices. Bioinsecticides like spinosad are increasingly recognized for their reduced environmental impact compared to synthetic pesticides. By providing a reliable method for monitoring bioinsecticide concentrations in soil, this study contributes to the safe and effective use of bioinsecticides within integrated pest management (IPM) programs. The high accuracy, precision, and sensitivity of the qNMR method make it a valuable tool for both environmental monitoring and regulatory compliance, supporting sustainable pest management practices and ensuring agricultural productivity while protecting environmental health.

## Experimental

### Materials and reagents

The experimental phase of this study utilized equipment and materials from the Organic Chemistry Laboratory at Campus III of the Instituto de Ecología A.C. (INECOL) in Xalapa, Veracruz, Mexico. The commercial bioinsecticide product SPINTOR 12SC® was used as the source for spinosad extraction. According to the product label, SPINTOR 12SC® contains 11.6% w/v of spinosad in a 250 mL solution. Based on this concentration, the total amount of spinosad used in the extraction process was calculated, ensuring accurate quantification for subsequent analysis.

All reagents used, including solvents such as deuterated chloroform (CDCl_3_) for NMR analysis and the internal standard 1,3,5-trimethoxybenzene (TMB), were of analytical grade and sourced from reputable suppliers (Sigma Aldrich) to maintain the precision and reliability of the experimental results.

### Instrumentation

#### High-performance liquid chromatography (HPLC)

HPLC equipped with a UV detector set at 250 nm was used to separate spinosyns A and D from the purified spinosad compound. A reverse-phase C18 column (250 mm × 4.6 mm, 5 μm particle size) was employed for the separation, using a specific elution gradient of water and acetonitrile. The total run time for each sample was 120 minutes, ensuring complete separation of the analytes. The detailed elution gradient conditions are provided in Table 1. The flow rate was maintained at 1.0 mL min^−1^, and the column temperature was set to 25 °C to ensure optimal separation conditions.

**Table 1:** Elution gradient used for the HPLC separation of spinosyns A and D

Time (min)	% Water	% Acetonitrile
5	30	70
110	1	99
5	30	70

#### NMR spectroscopy

NMR spectroscopy was performed using a Bruker BioSpin 500 MHz NMR spectrometer equipped with a Z119470_0187 (PA BBO 500S1 BBF-H-D-05 Z SP) probe for ^1^H and ^13^C NMR experiments. A Varian VNMRS spectrometer with a OneProbe was used for additional ^13^C NMR measurements. All samples were dissolved in deuterated chloroform (CDCl_3_) for analysis, and experiments were conducted at temperatures ranging from 292.7 K to 298.1 K.

^1^H NMR Spectra were acquired using the standard ‘zg30’ pulse sequence with a pulse width of 11.050 μs and a relaxation delay (*D*_1_) of 1 second. A total of 16 scans were performed per sample, with a spectral width of 10 000 Hz. The acquired data size was 32 768 points, and zero-filling was applied to expand the spectral size to 65 536 points for improved digital resolution.

For ^13^C NMR, ‘s2pul’ pulse sequence was used, with a pulse width of 5.3375 μs and a relaxation delay of 1 second. A total of 5000 scans were collected to ensure sufficient signal for ^13^C nuclei, with a spectral width of 31 250 Hz.

For COSY, proton–proton couplings were determined using the ‘cosygpppqf’ pulse sequence, with 2 scans and a relaxation delay of 2 seconds. The spectral width was set to 3906.2 Hz for both dimensions.

For HSQC, direct proton–carbon correlations were identified using the ‘hsqcedgpph’ pulse sequence, with 2 scans and a relaxation delay of 1.5 seconds. The spectral width was 3705.5 Hz for ^1^H and 22 321.4 Hz for ^13^C.

For HMBC, long-range heteronuclear couplings were examined using the ‘hmbcgpndqf’ pulse sequence, with 4 scans and a relaxation delay of 1.5 seconds. The spectral width was 3705.5 Hz for ^1^H and 26 041.7 Hz for ^13^C.

The NMR data were processed using zero-filling to enhance the resolution. Additional processing steps included baseline correction, apodization, and phase adjustments as needed. In the HSQC spectra, only positive peaks were selected for analysis. This approach was chosen because positive peaks correspond to direct one-bond correlations between ^1^H and ^13^C nuclei, providing the most straightforward and relevant structural information.

#### Purification and identification of spinosad

The purification of spinosad was a critical step to ensure the accuracy and reliability of the qNMR method for soil residue analysis. The commercial product SPINTOR 12SC®, containing spinosad as the active ingredient, includes formulation agents that could interfere with NMR signals. Purification was necessary to isolate spinosyns A and D, the active components of spinosad, and establish a baseline for quantification. Liquid–liquid extraction was performed by mixing the commercial product with ethyl acetate and brine to separate spinosad from water-soluble impurities, followed by drying the organic layer over sodium sulfate. Thin-layer chromatography (TLC) confirmed the presence of spinosad, using a solvent system of AcOEt/MeOH (9 : 1) for optimal resolution. Final purification employed column chromatography with a gradient of AcOEt/MeOH (95 : 05) to obtain spinosad in crystalline form, ensuring high purity for analyses. The purity of the isolated compound was verified using ^1^H and ^13^C NMR and HPLC for spinosyn A and D separation.

To assess the method's accuracy and precision, soils were spiked with known quantities of spinosad. Two soil types were used: red loamy soil from Veracruz, Mexico, and commercially available black organic soil (Nutrigraden®) from Querétaro, Mexico. The soils were air-dried, pulverized, and sieved to ensure uniform particle sizes. Spinosad in acetone was evenly sprayed onto 10 g soil aliquots, followed by manual mixing for homogeneity. The spiked soils were left under a fume hood for 24 hours to evaporate acetone, leaving spinosad residues. Unspiked soils served as controls to monitor background signals.

Spiked soils were subjected to a three-step extraction process with a solvent system optimized for spinosad recovery. Recovery and precision were evaluated by analyzing replicates at varying concentrations (2 mg kg^−1^, 5 mg kg^−1^, and 8 mg kg^−1^). This process validated the qNMR method's performance, ensuring its applicability for routine environmental monitoring and regulatory compliance.

#### Selection of internal standard

Various compounds traditionally used as internal standards were evaluated for their non-interference in the spectral region of interest, solubility in CDCl_3_, stability, and accessibility. The selection was based on obtaining ^1^H NMR spectra for each candidate, followed by quantification calculations and standard deviation analysis. The chosen internal standard, TMB, did not interfere with the spinosad signals and provided favorable calculation outcomes.

#### Quantitative nuclear magnetic resonance (qNMR)

The quantification of spinosad was conducted using ^1^H NMR, where the spectra of spinosyns A and D were analyzed to calculate the mass of the active ingredient in relation to the internal standard (TMB). The calculation was based on the following equation:
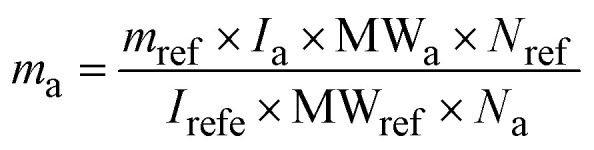
*m*_a_ = mass in g of the active spinosad (g) *m*_ref_ = mass in g of the internal standard (g) *I*_a_ = integrated area of the spinosad signal *I*_ref_ = integrated area of the internal standard (TMB) signal MW_a_ = molecular weight of the spinosad (g mol^−1^) MW_ref_ = molecular weight of the internal standard (TMB) (g mol^−1^) *N*_ref_ = number of equivalent nuclei (protons) contributing to the signal of the internal standard (TMB). *N*_a_ = number of equivalent nuclei (protons) contributing to the signal of the spinosad.

This equation allows the precise quantification of spinosad by comparing the areas of the analyte and internal standard signals, considering their respective molecular weights and proton equivalence. By using ^1^H NMR, the method ensures that quantification is accurate and reproducible, with minimal sample preparation required.

To validate the method, the analysis was conducted using four independent replicates of Spintor 12SC®. The measured spinosad concentration averaged 11.58 ± 0.33%, aligning closely with the manufacturer's stated concentration of 11.6%.

#### Optimization of parameters

NMR spectrometer calibration followed the manufacturer's guidelines, optimizing spectral peak resolution and calculating relaxation times to determine the appropriate repetition time between pulses for the sample analysis.

#### Laboratory soil sample quantification tests

The study utilized two distinct soil types for analysis. The first sample was a red soil collected from the southern region of Veracruz, Mexico, at coordinates 18.0152369, −94.5797643. The second sample was a commercially available black soil branded as Nutrigraden® from Querétaro, Mexico. A solid–liquid extraction was initially performed using only solvent to obtain a ^1^H NMR spectrum, which served as the blank reference for the soil samples. The red soil sample collected from Veracruz has a slightly acidic pH and a loamy texture, which is typical for the region's agricultural land. The commercially obtained black soil from Nutrigraden® is characterized by its higher organic content and neutral pH, making it representative of garden soils in Querétaro.

Soil preparation followed a meticulous process to ensure sample homogeneity before bioinsecticide impregnation. Each soil sample was dried, pulverized, and sieved to remove large particulates, creating a uniform matrix for consistent analysis. This preparation step was crucial to achieve an even distribution of spinosad throughout the sample, facilitating consistent extraction and quantification.

To maximize spinosad recovery, an iterative extraction process was employed, with a carefully selected solvent chosen for its efficiency in extracting spinosad without interfering with its NMR signals, thereby preserving quantification accuracy. The extraction was repeated three times to ensure optimal bioinsecticide recovery.

The two soil types were compared to assess the method's adaptability to various agricultural environments. The soil type yielding the most reliable and reproducible NMR spectra was selected for further analysis, underscoring the method's flexibility across different soil compositions.

Following extraction, the samples were analyzed using ^1^H NMR spectroscopy to quantify the recovered spinosad. This multi-step process—from soil preparation to NMR analysis—demonstrates the robustness and precision of the method in detecting and quantifying bioinsecticides in soil, supporting its potential for routine environmental monitoring and agricultural applications.

#### Method validation

The method was validated to ensure its reliability and precision in analyzing spinosad in soil samples. The validation parameters included specificity, linearity, sample stability, precision, accuracy, and limits of detection (LOD) and quantification (LOQ).

Specificity was evaluated by assessing potential interferences between the solvent (CDCl_3_), the internal standard (TMB), and the analyte (Spinosad). Reference mixture samples were analyzed, and no cross-signals or interferences were observed, confirming that the method can accurately detect spinosad without interference from other components.

Linearity was established by constructing a calibration curve using dilutions of a 10 mg mL^−1^ spinosad solution in CDCl_3_ with TMB as the internal standard. The calibration curve was defined by the equation: *y* = 2251*x* + 6800.

The regression coefficient (*R*^2^) was 0.9928, indicating a strong linear relationship, and the standard error of the estimate was 463.841. The linearity range for the method was 2–8 mg mL^−1^.

Accuracy and precision were evaluated through intraday and interday analyses at concentrations of 3 mg mL^−1^ and 6 mg mL^−1^. For both analyses, three replicate measurements (*n* = 3) were performed per concentration. The method showed high accuracy, with a mean error of −0.56% for the 3 mg mL^−1^ concentration and 2.76% for the 6 mg mL^−1^ concentration. Precision was demonstrated by low standard deviations, with intraday standard deviations of 0.022 mg mL^−1^ for 3 mg mL^−1^ and 0.039 mg mL^−1^ for 6 mg mL^−1^. The coefficients of variation (CV) were 0.74% and 0.63%, respectively, reflecting the method's reproducibility. Statistical validation was performed using one-way ANOVA, confirming no significant variation between replicates (*p* > 0.05), thereby validating the precision and reproducibility of the method.

The LOD and LOQ were determined from serial dilutions of a 6 mg mL^−1^ stock solution of spinosad. The LOD and LOQ were calculated using the following equations:
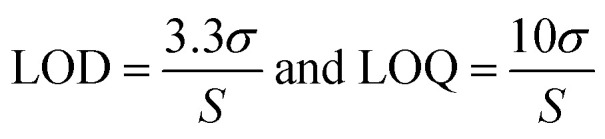
where, *σ* is the standard deviation of the response (28.23), *S* is the slope of the calibration curve (2251).

The LOD was determined to be 0.0414 mg mL^−1^, and the LOQ was 0.1254 mg mL^−1^, indicating that the method is sensitive enough to detect and quantify low concentrations of spinosad.

Solution stability was assessed over a period of 28 hours. Samples were analyzed at 0, 4, 8, 24, and 28 hours to evaluate any potential degradation of spinosad. The concentration remained stable at 0.5 mg mL^−1^ throughout the testing period, demonstrating that the spinosad solution is stable for at least 28 hours under the tested conditions.

## Data availability

The data supporting this article have been included as part of the ESI.†

## Author contributions

T. J. P.: methodology, data curation, investigation, software, writing – original draft, writing – review & editing; S. X. R. C.: methodology, investigation, software, writing – original draft; I. B. L.: methodology, investigation; G. M.: resources, writing – review & editing; E. D. A.: resources, writing – review & editing; S. V. P.: resources, writing – review & editing; I. D. P. L.: conceptualization, investigation, supervision, writing – review & editing; J. L. O. R.: conceptualization, funding acquisition, investigation, project administration, resources, supervision, validation, writing – review & editing.

## Conflicts of interest

There are no conflicts to declare.

## Supplementary Material

RA-015-D5RA00356C-s001
