# Supplementary material for: Quantitative NMR for detection of spinosad residues in agricultural soils
Source: RSC Adv. 2025 Feb 18;15(7):5547–57. doi: 10.1039/d5ra00356c (PMC11834155; doi:10.1039/d5ra00356c)

Supplementary Information (SI) for RSC Advances.
This journal is © The Royal Society of Chemistry 2025

Supporting Information

Quantitative NMR for Detection of Spinosad Residues in Agricultural Soils

Tushar Janardan Pawar,[a] Siuly Xenia Ramos-Cruz,[a,b] Israel Bonilla-Landa,[a] Ghazala Muteeb,[c] Enrique Delgado-Alvarado,[d] Sachin V. Patil,[e] Irving David Perez-Landa,[b] and José Luis Olivares-Romero.*[a]

a. *Red de Estudios Moleculares Avanzados, Instituto de Ecología, A.C., Carretera Antigua a Coatepec 351, Xalapa, 91073, Veracruz, México..*
b. *Tecnológico Nacional de México/Instituto Tecnológico de Boca del Río, Carretera Veracruz-Córdoba 12, Boca del Río, 94290, Veracruz México..*
c. *Department of Nursing, College of Applied Medical Sciences, King Faisal University, Al-Ahsa, Saudi Arabia.*
d. *Micro and Nanotechnology Research Center, Universidad Veracruzana, Blvd. Av. Ruiz Cortines No. 455 Fracc. Costa Verde, Boca del Río, Veracruz 94294, México.*
e. *Department of Chemistry, Research Centre HPT Arts and RYK Science College (Affiliated to S. P. Pune University), Nashik, 422005, Maharashtra, India.*

jose.olivares@inecol.mx

Table of Contents

1. General Information S2
2. Graphical Data and Statistical Analysis S3
3. Soil samples S10
3. HPLC Chromatogram S12
4. NMR spectra S13

1. General Information

All chemicals and reagents used in this study, including Spinosad (SPINTOR 12SC®), ethyl acetate (AcOEt), methanol (MeOH), sodium sulfate (Na_2SO_4), and all solvents, were of analytical grade and were purchased from Sigma Aldrich. The commercial Spinosad formulation, SPINTOR 12SC®, was sourced directly from authorized distributors.

The HPLC system used in this study was equipped with a UV detector set at 250 nm for the separation and identification of Spinosyns A and D. A reverse-phase C18 column was utilized for the chromatographic separation, and the mobile phase consisted of a gradient elution of water and acetonitrile. The specific elution gradient conditions are detailed in Table 1 of the manuscript. The total run time for each sample was 120 minutes, allowing for precise separation of the bioinsecticide components.

A Bruker Advance HD 500 MHz NMR spectrometer was employed to conduct various NMR experiments, including ^{1}H, ^{13}C, COSY, HMBC, and HSQC. The Spinosad samples were dissolved in deuterated chloroform ($CDCl_3$) for analysis. Several compounds were evaluated as internal standards, including tetramethylsilane (TMS), benzoic acid (BA), and 1,3,5-trimethoxybenzene (TMB). TMB was selected for its non-interference with Spinosad signals and favorable quantification results.

All graphs and statistical analyses were created using the Python program.

NMR-Instrumental details:

Parameter	1D Proton (1H)	1D Carbon-13 (13C)	2D COSY	2D HSQC	2D HMBC
**Pulse Sequence**	zg30	s2pul	cosygpppqf	hsqcedgpph	hmbcgpndqf
**Number of Scans**	16	5000	2	2	4
**Relaxation Delay (D1)**	1.0000 seconds	1.0000 seconds	2.0000 seconds	1.5000 seconds	1.5000 seconds
**Pulse Width**	11.0500 µs	5.3375 µs	10.0000 µs	11.0500 µs	11.0500 µs
**Spectral Width**	10,000 Hz	31,250 Hz	3906.2 Hz (F2, F1)	3705.5 Hz (1H), 22321.4 Hz (13C)	3705.5 Hz (1H), 26041.7 Hz (13C)
**Acquired Size**	32,768 points	32,768 points	1024 points (F2), 128 points (F1)	512 points (F2), 256 points (F1)	2048 points (F2), 256 points (F1)
**Spectral Size**	65,536 points	65,536 points	1024 points (F2), 1024 points (F1)	512 points (F2), 512 points (F1)	2048 points (F2), 1024 points (F1)

2. Graphical Data and Statistical analysis:

A) Accuracy and Precision

This section presents the accuracy and precision data for the qNMR method used to quantify Spinosad. It includes the nominal concentration, mean measured concentration, standard deviation, coefficient of variation, and mean error for two concentrations (3 mg/mL and 6 mg/mL) (Table S1).

Table S1: Accuracy and Precision of the qNMR Method for Spinosad.

Concentration (mg/mL)	Mean Measured Concentration (mg/mL)	Standard Deviation (mg/mL)	Coefficient of Variation (%)	Mean Error (%)
3	2.983	0.022	0.74	-0.56
6	6.155	0.039	0.63	2.76

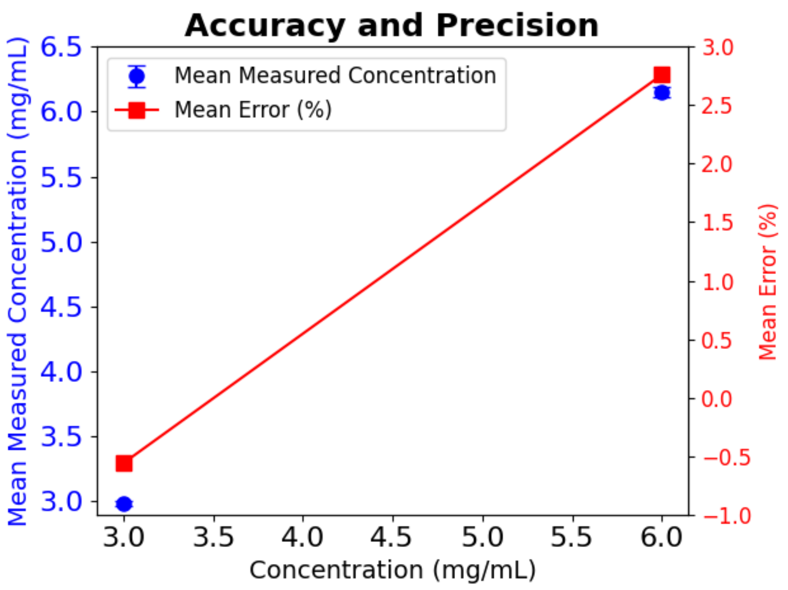

Figure S1: Accuracy and Precision of the qNMR Method for Spinosad.

The Figure S1 illustrates the accuracy and precision of the qNMR method used for Spinosad quantification. The blue circles represent the mean measured concentrations at nominal concentrations of 3 mg/mL and 6 mg/mL, while the red squares show the mean error (%) for these concentrations. The left y-axis corresponds to the mean measured concentration, and the right y-axis corresponds to the mean error (%). The data points indicate high precision and accuracy of the method, as evidenced by the small standard deviations and low mean error values.

B) Calibration Curve

The Table S2 shows the calibration data for various concentrations of Spinosad. For each concentration (ranging from 2 mg/mL to 8 mg/mL), the values for three replicates are provided, along with their average.

Table S2: Calibration Data for Spinosad Concentrations. The table shows the NMR signal intensities for various concentrations of Spinosad (ranging from 2 mg/mL to 8 mg/mL) measured in triplicates. Each value represents the area under the specific NMR signal peaks corresponding to Spinosad. The average signal intensity for each concentration is also provided.

**Concentrations**	**8 mg/mL**	**6 mg/mL**	**5 mg/mL**	**4 mg/mL**	**3 mg/mL**	**2 mg/mL**
1	24615.3	20789.3	19952.6	16458.3	13452.7	11120.6
2	23944.4	20172.7	17933.1	16140.4	13393.4	10621
3	24308.4	21142.1	17159.5	15826.2	13745.2	10711
**Average**	**24289.37**	**20701.37**	**18348.4**	**16141.63**	**13530.43**	**10817.53**

Limit of Detection and Quantification:

The limit of detection (LOD) and limit of quantification (LOQ) were determined through serial dilutions of a stock solution of Spinosad (6 mg/mL). The LOD was identified at 0.04 mg/mL, and the LOQ at 1.2 mg/mL, showcasing the method's sensitivity. The LOD and LOQ were calculated based on the standard deviation of the response and the slope of the calibration curve using the formulas:

$$LOD = \frac{3.3\sigma}{S}$$

$$LOQ = \frac{10\sigma}{S}$$

Where,

σ is the standard deviation of the response, and

S is the slope of the calibration curve.

Table S3: Limit of Detection and Quantification Data. This table presents the NMR signal intensities for different dilutions of a stock solution of Spinosad (6 mg/mL) used to determine the limit of detection (LOD) and limit of quantification (LOQ). The intensities are the areas under the NMR signal peaks corresponding to Spinosad at each dilution level. The calculated LOD and LOQ values are based on these intensities.

**Sample**	**Concentration (mg/mL)**	**Intensity**
Stock Solution	6	141.7
Dilution 1:5	1.2	87.8
Dilution 1:10	0.6	75.7
Dilution 1:25	0.24	44.8
Dilution 1:40	0.15	34.4
Dilution 1:50	0.12	25.6
Dilution 1:100	0.06	17.4
Dilution 1:150	0.04	15.5
Dilution 1:200	0.03	0

Residual Analysis of the Calibration Curve:

The residuals of the calibration curve were analyzed to validate the fit of the linear model. The residuals are the differences between the observed and predicted values of the area under the curve for each concentration.

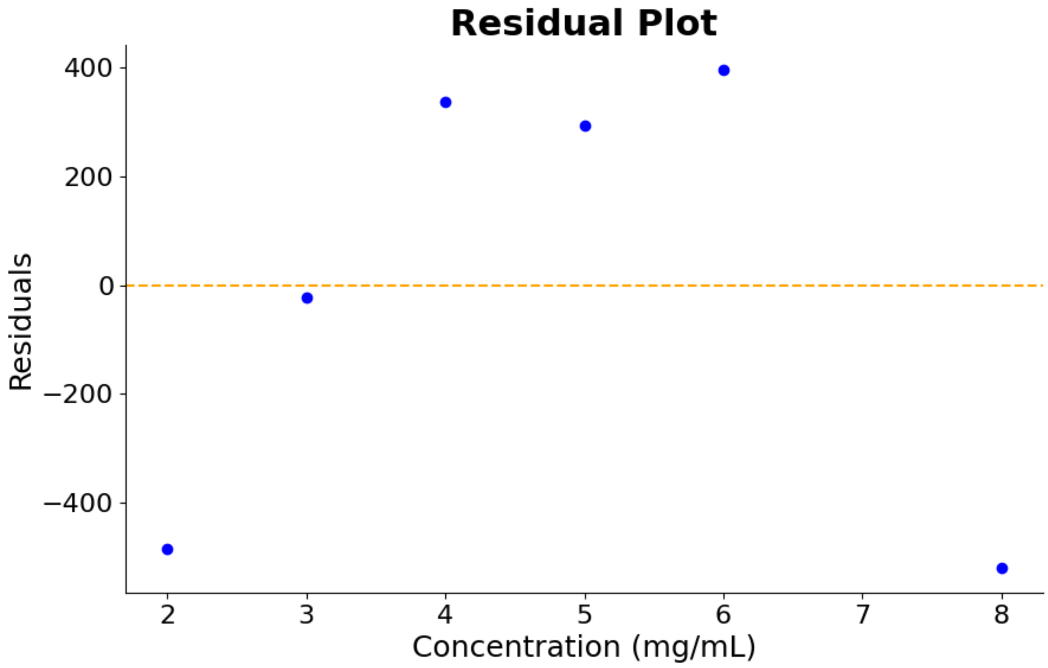

Figure S2. Residual plot showing the differences between observed and predicted values for each concentration.

The residual plot indicates that the residuals are randomly distributed around zero, suggesting that the linear model is appropriate for the data. The standard error of the estimate is also provided to quantify the accuracy of the predictions.

Standard Error of the Estimate:

The standard error of the estimate was calculated as follows:

$$Standard\ Error = \sqrt{\frac{\sum(y_i - \hat{y}_i)^2}{n-2}}$$

Where,

y_i are the observed values

$\hat{y}_i$ are the predicted values and

n is the number of data points

Solution stability:

Solution stability was verified over a period of 28 hours. Samples were analyzed at time intervals of 0, 4, 8, 24, and 28 hours to determine the stability of the Spinosad solution. The concentration of Spinosad remained consistent at 0.5 mg/mL throughout the testing period, indicating no significant degradation. The stability tests were performed at controlled temperatures and protected from light, affirming the method's reliability over time.

Table S4: Solution Stability Data

Time (hours)	Concentration (mg/mL)
0	0.5
4	0.5
8	0.5
24	0.5
28	0.5

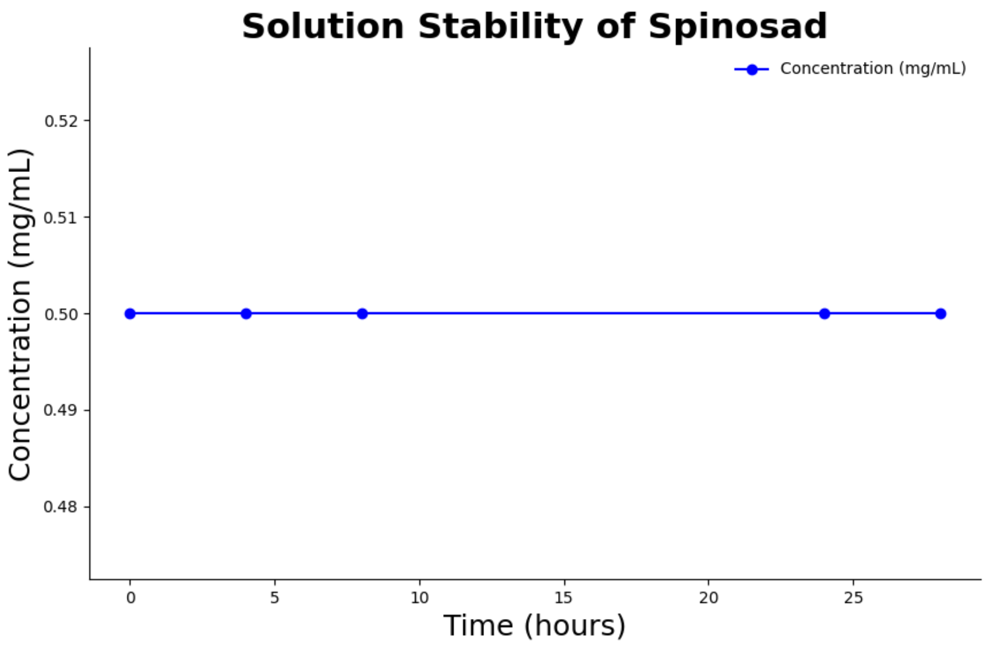

Figure S3: Solution Stability Plot.

Calculated values:

Slope, m = 2251

Intercept, c = 6800

Regression Coefficient, R^2 = 0.99

Standard error = 463.841

3. Soil samples

This study employed two distinct soil types to evaluate the qNMR method's adaptability across different soil compositions. Detailed information on the soil samples is provided below, along with representative images to illustrate the physical characteristics of each soil type.

3.1 Red Soil from Veracruz

The first soil sample was a red soil collected from the southern region of Veracruz, Mexico, specifically from a location near Minatitlán at coordinates 18.0152369, -94.5797643. This soil is representative of agricultural land in the region, characterized by its slightly acidic pH and loamy texture, which supports diverse crop types. The red color is indicative of iron oxide content, which may influence soil properties and interactions with Spinosad. The collected soil was air-dried, pulverized, and sieved to ensure a homogenous sample matrix prior to analysis. A picture of the red soil sample is provided below (Figure S4).

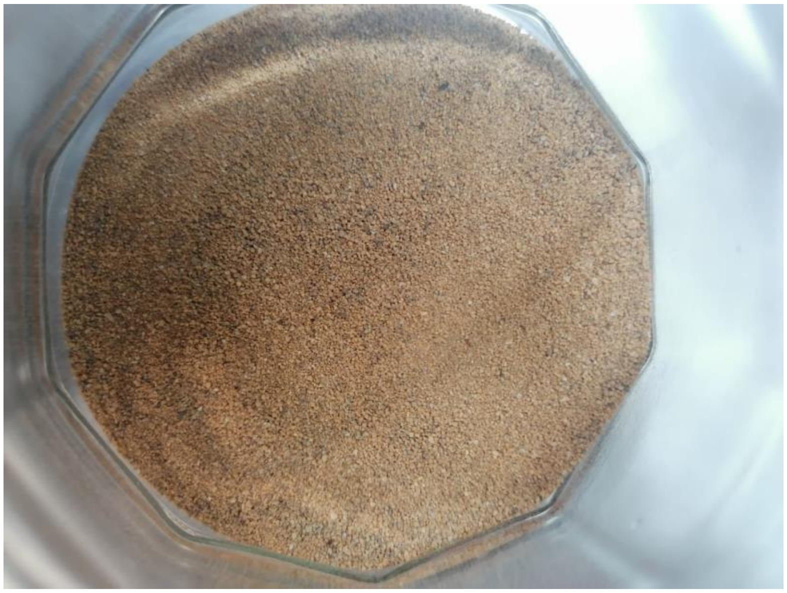

Figure S4: Red soil sample from Veracruz (near Minatitlán).

3.2 Black Soil (Nutrigraden®) from Querétaro

The second soil sample is a commercially available black soil branded as Nutrigraden®, sourced from Querétaro, Mexico. This soil is commonly used for gardening and is characterized by a higher organic matter content and a neutral pH, making it suitable for diverse horticultural applications. The black color indicates a high level of organic materials, which could affect the bioavailability and extraction of Spinosad. The soil underwent the same preparation process as the red soil, including drying, pulverizing, and sieving to ensure uniformity. An image of the black soil sample is included below (Figure S5).

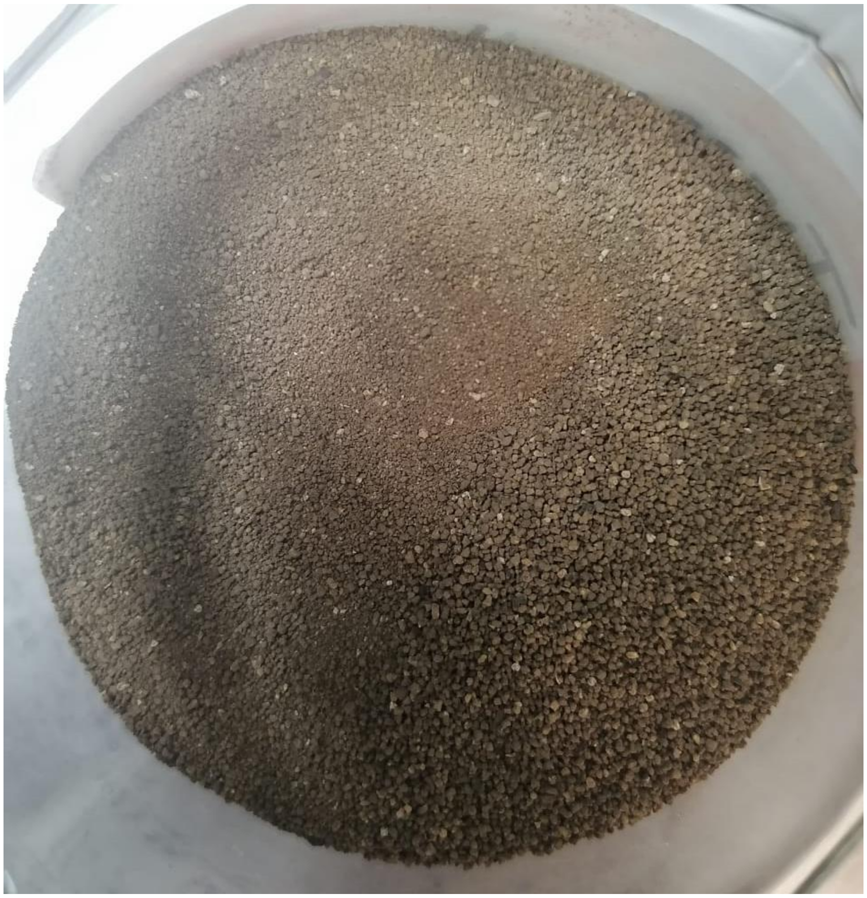

Figure S5: Black Nutrigraden® soil sample from Querétaro.

3.3 Sample Preparation and Consistency

Both soil samples were prepared meticulously to ensure consistency in Spinosad distribution and extraction efficiency. This preparation process included drying to remove moisture, grinding to achieve a fine particle size, and sieving to eliminate any larger debris. These steps were essential for creating a homogenous sample matrix that supports reliable and reproducible quantification results.

4. HPLC Chromatograms

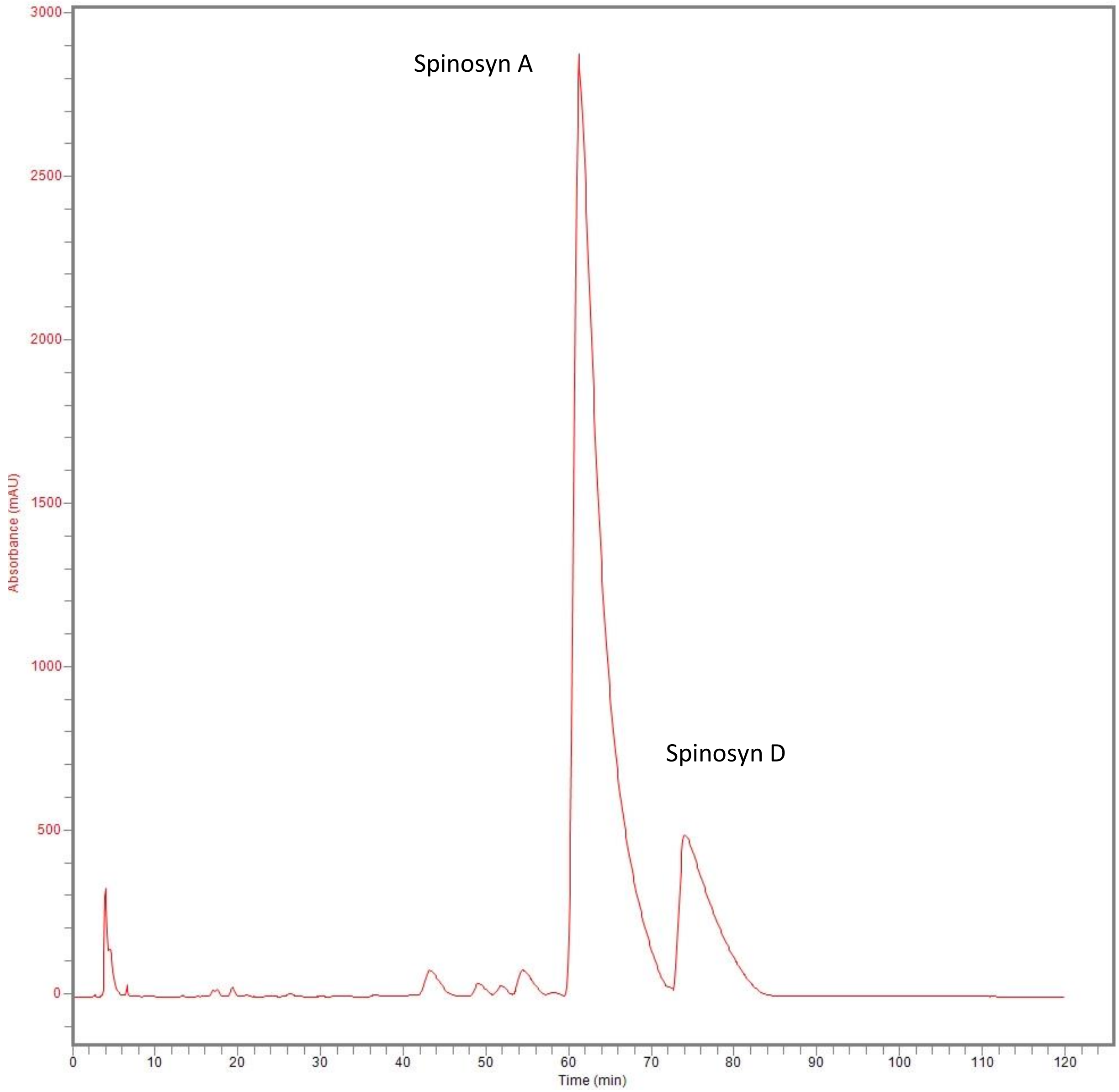

5. NMR spectra

^{1}H NMR spectra of Spinosad obtained from Spintor 12SC®

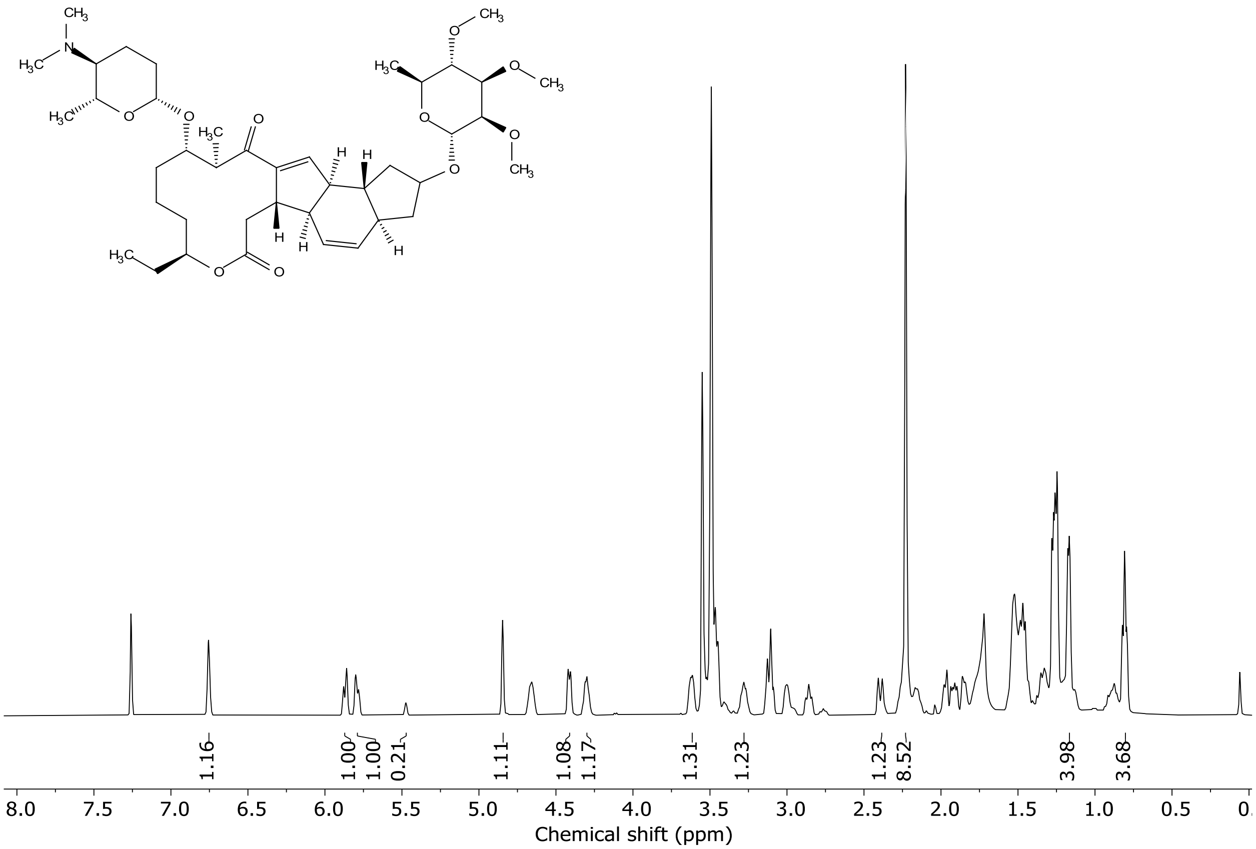

Comparation of the ^{1}H NMR spectra of Spinosad obtained from Gadeon 12SC® (green) and Spintor12SC® (purple)

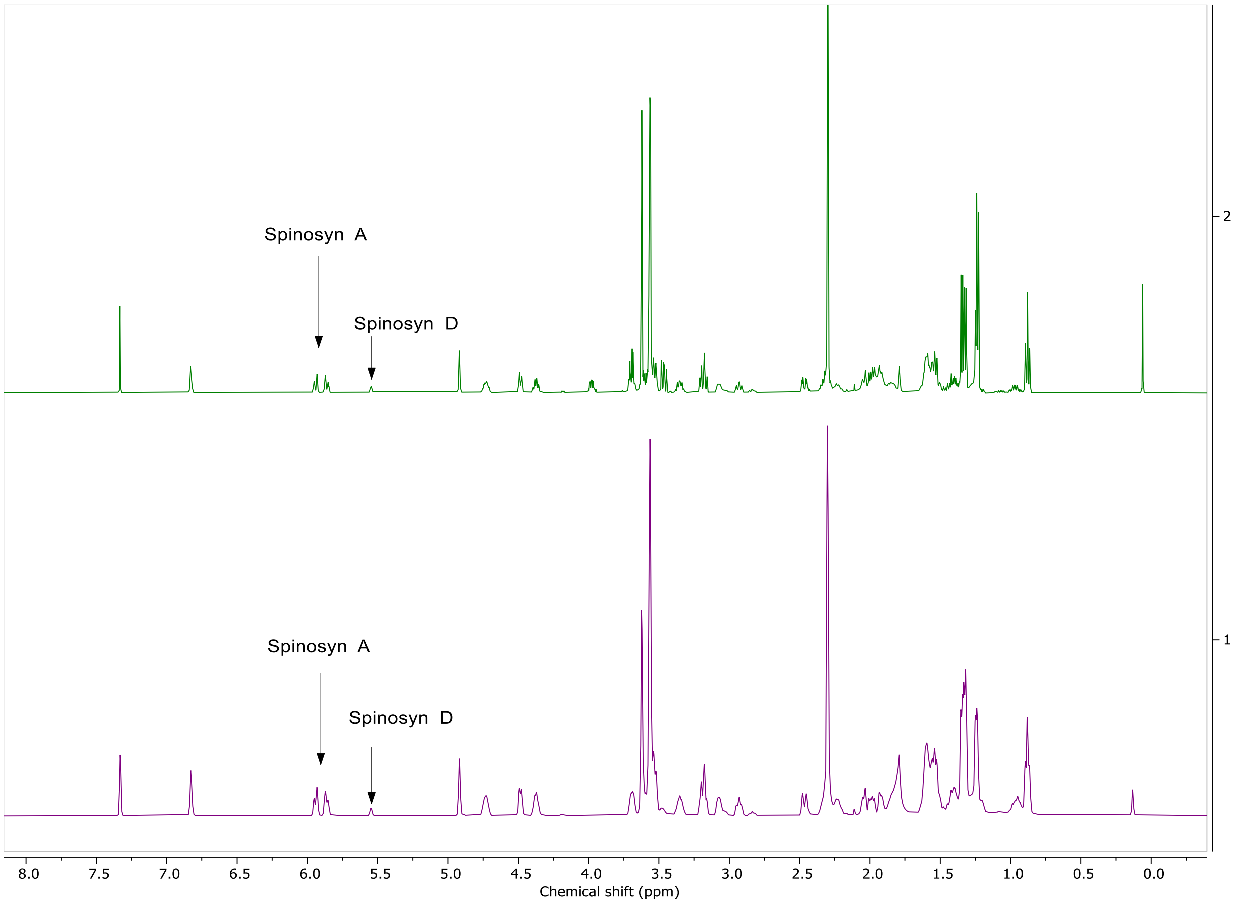

^{1}H NMR spectra of Spinosyn A

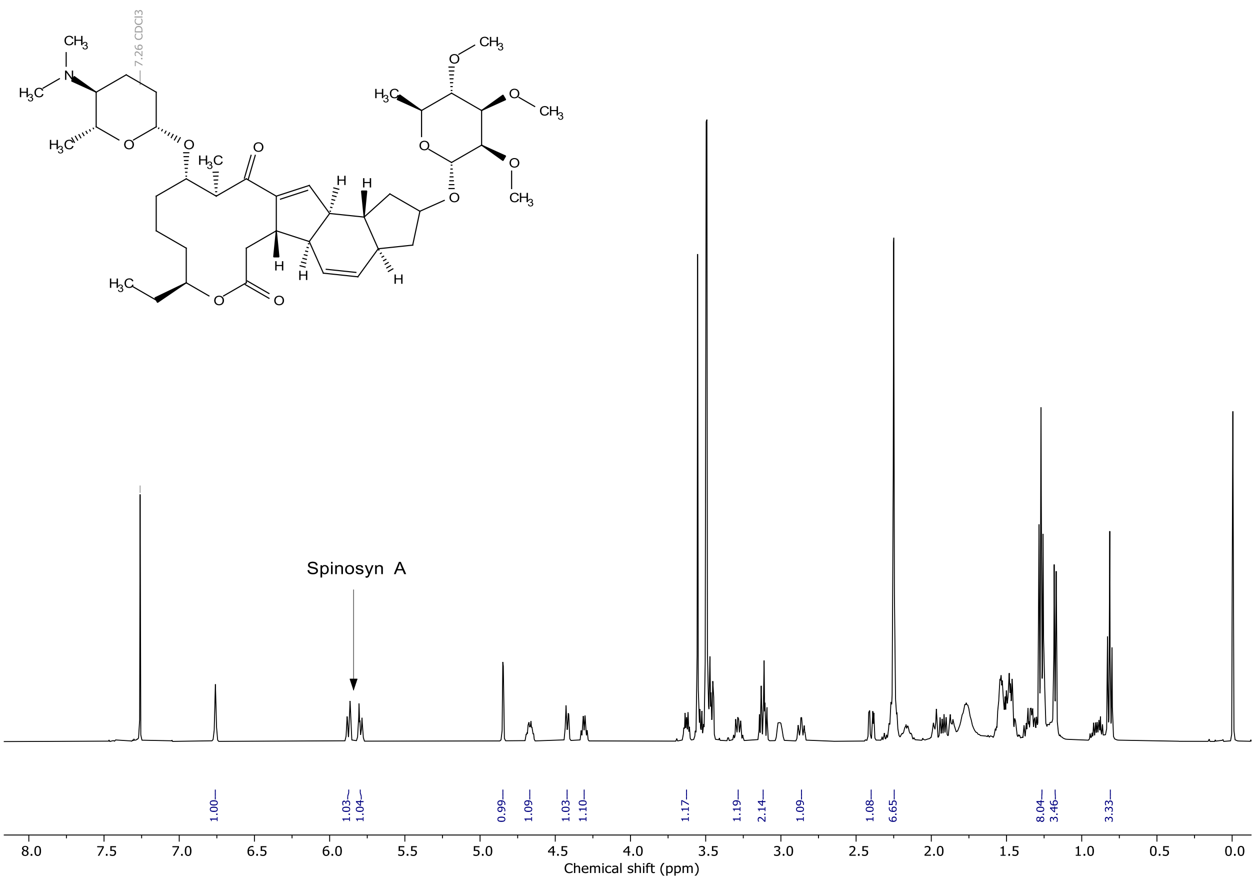

^{13}C NMR spectra of Spinosyn A

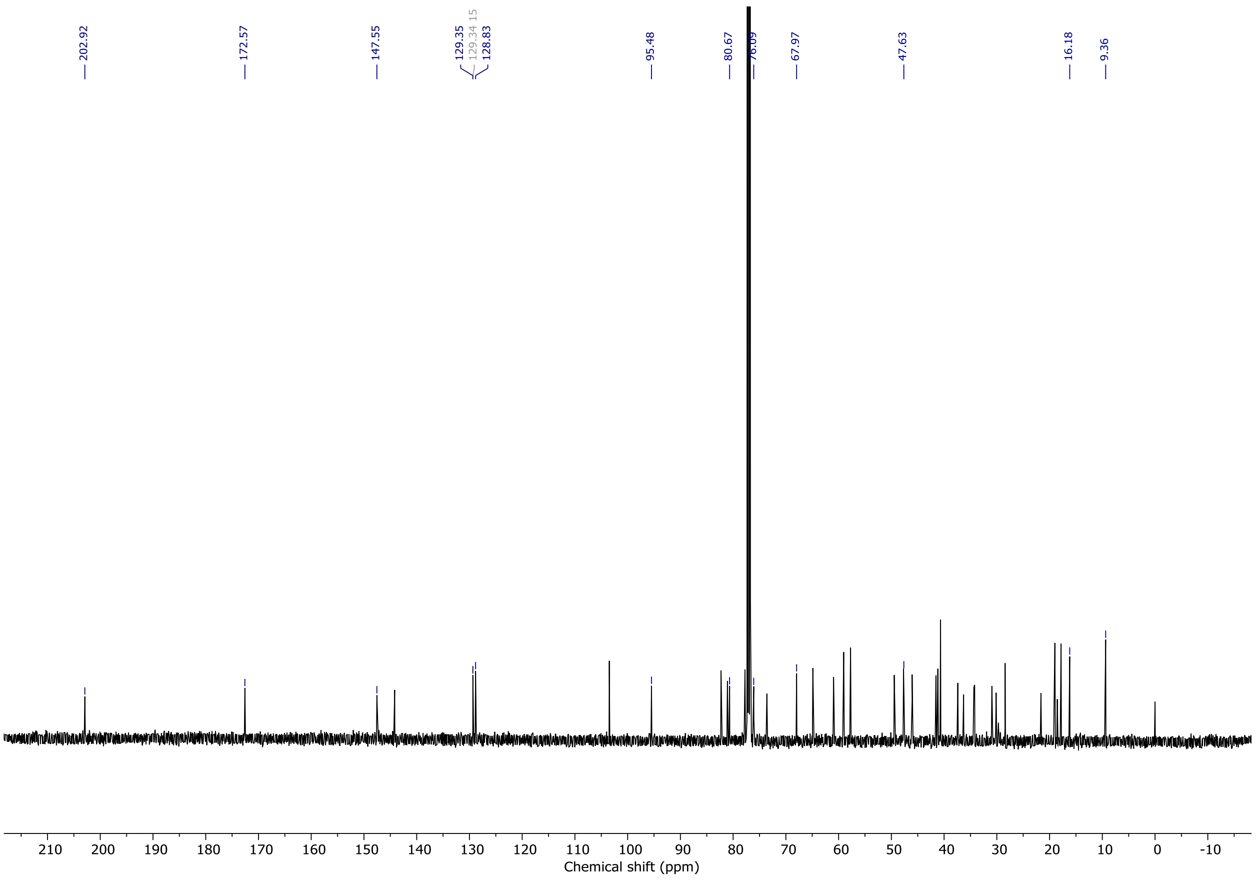

COSY NMR spectra of Spinosyn A showing the correlations between hydrogens

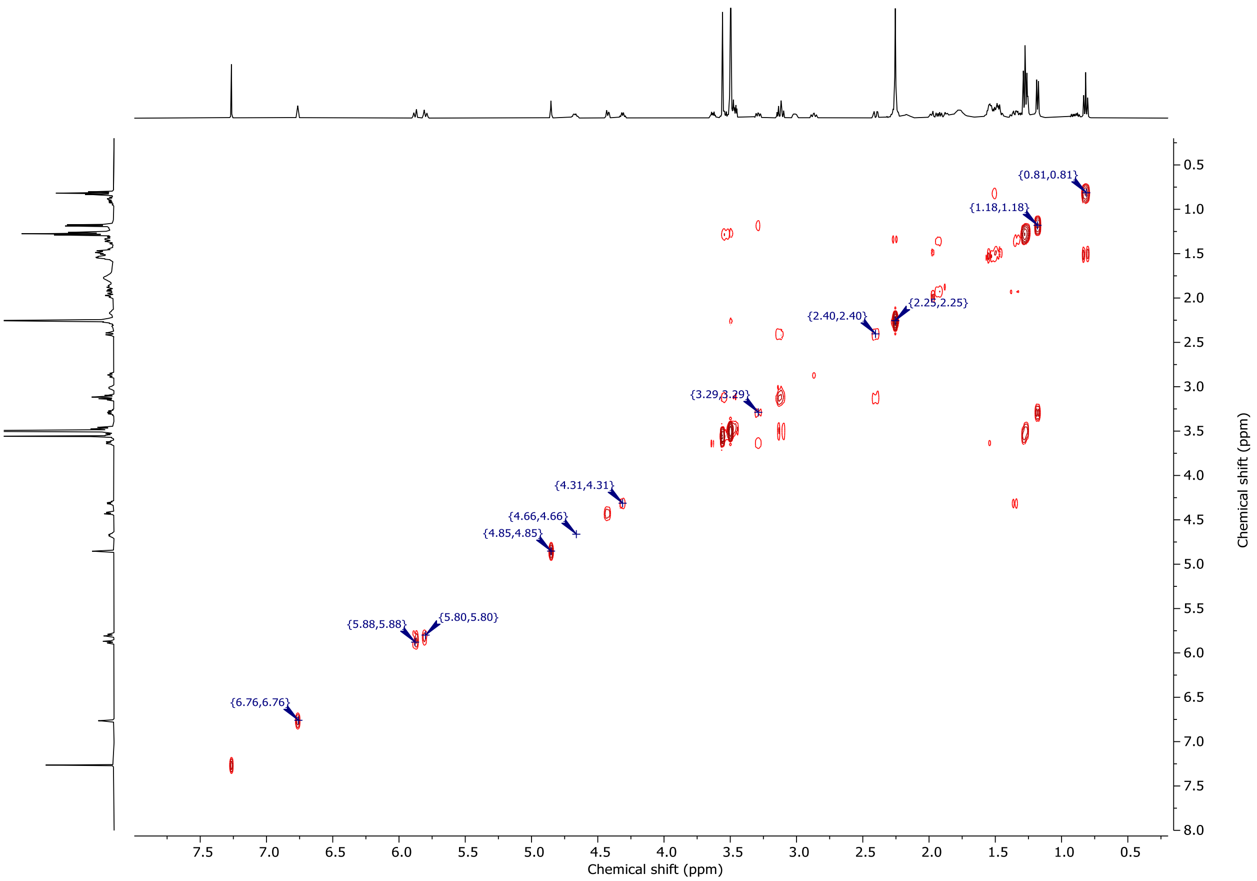

HSQC NMR spectra of Spinosyn A showing the correlations between hydrogen and carbon atoms

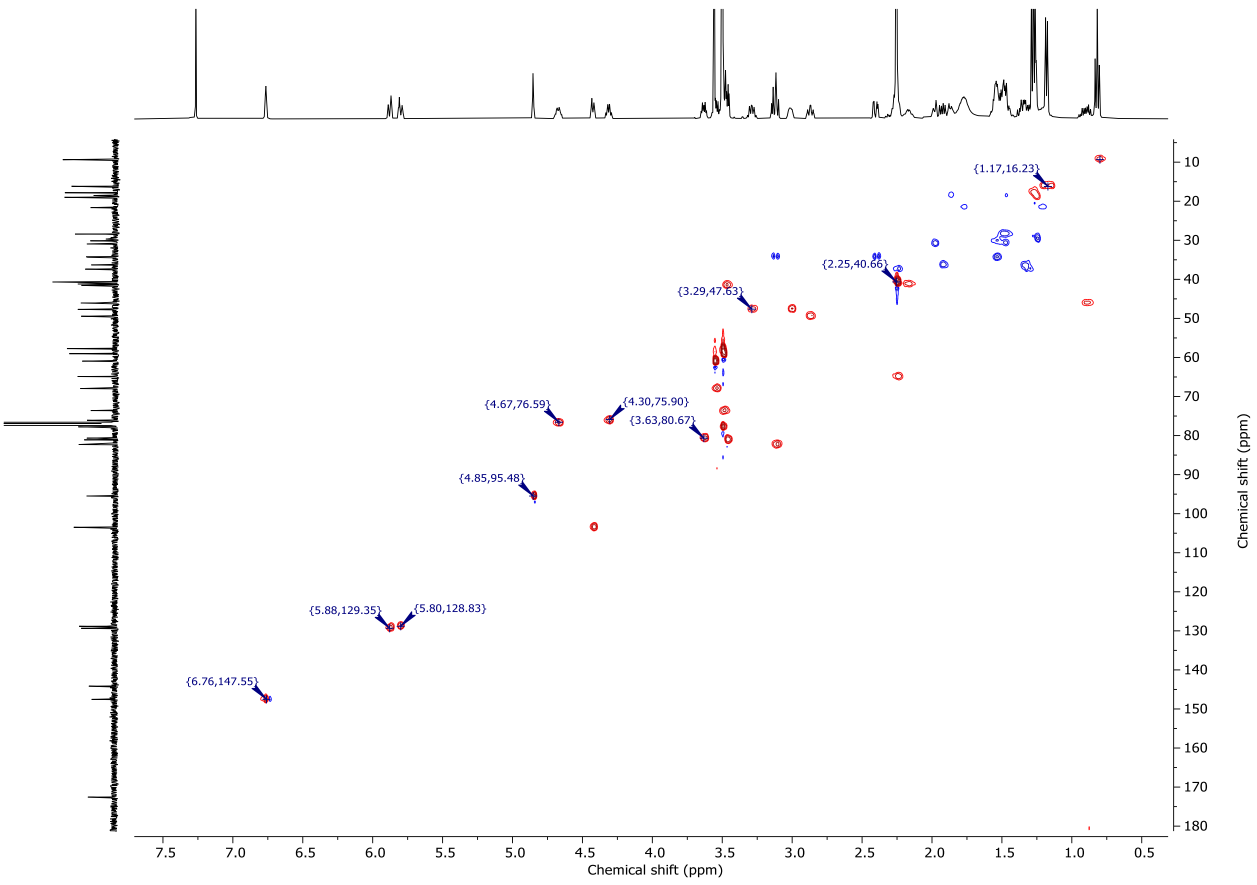

HMBC NMR spectra of Spinosyn A showing the correlations between hydrogen and carbon atoms

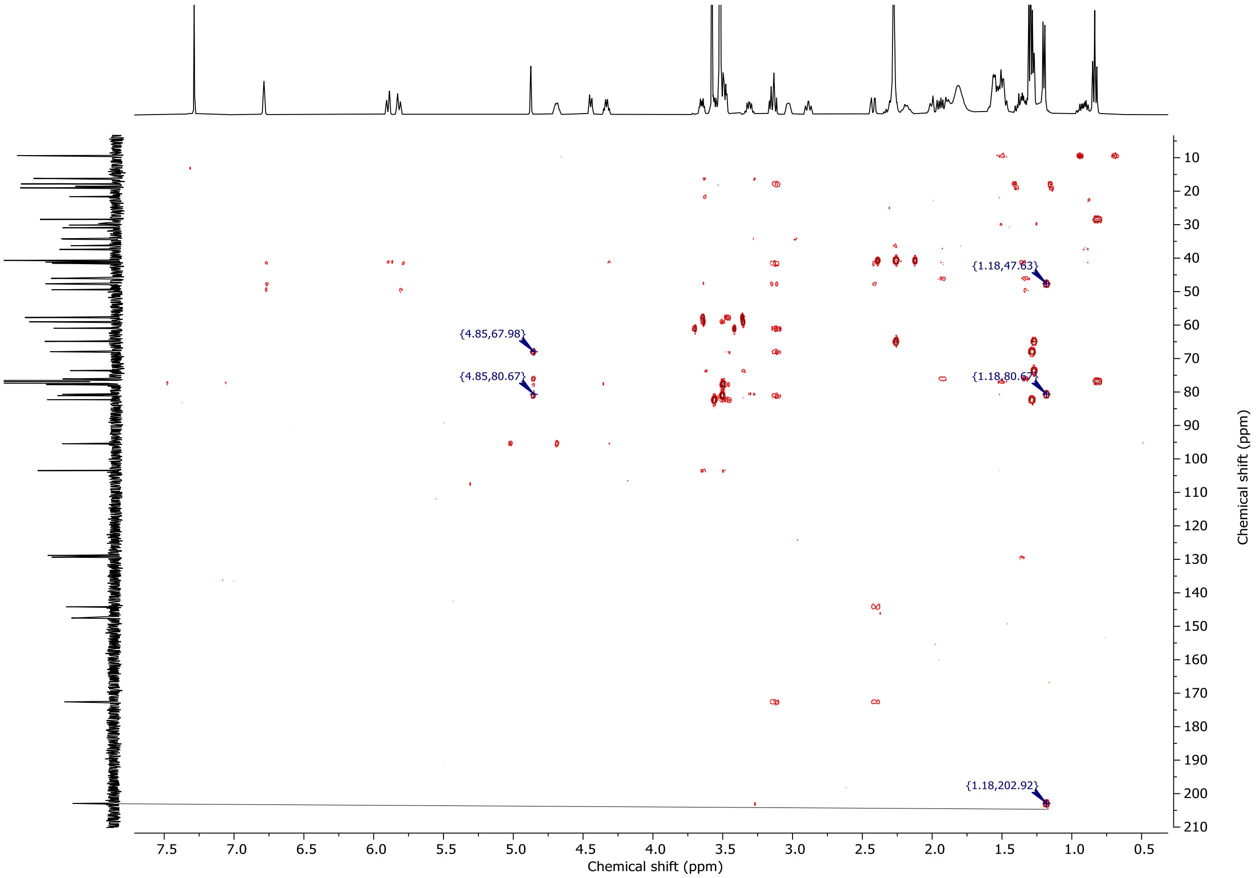

^{1}H NMR spectra of Spinosyn D

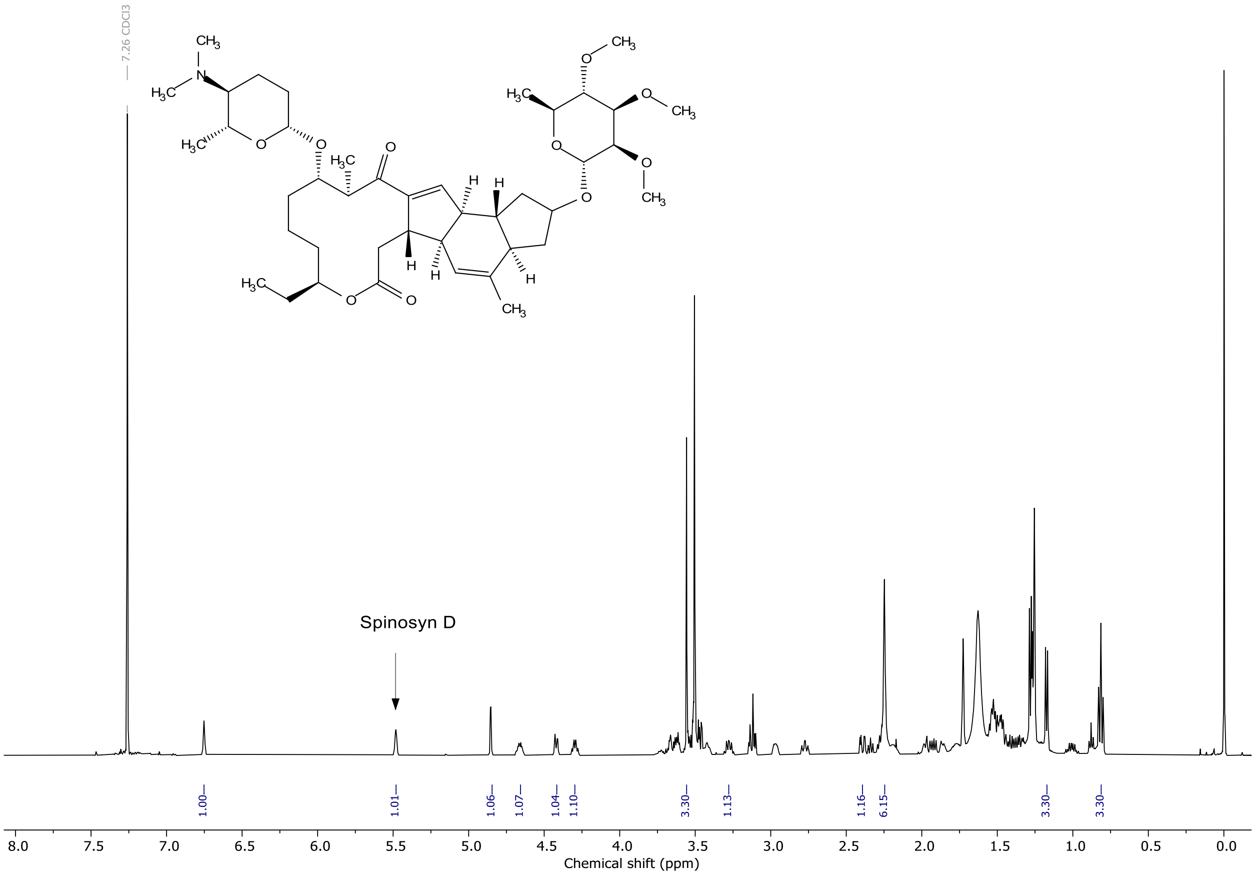

^{13}C NMR spectra of Spinosyn D

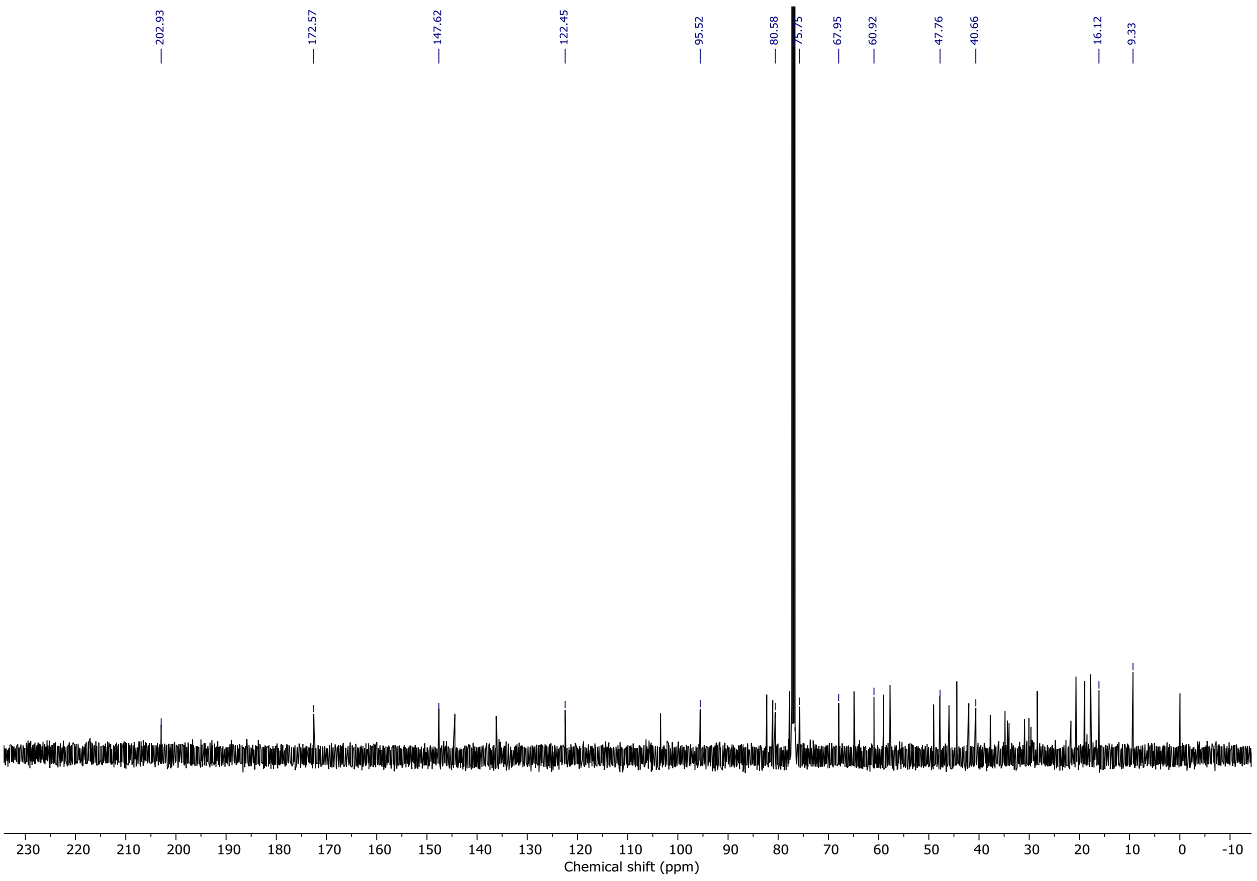

COSY NMR spectra of Spinosyn D showing the correlations between hydrogens

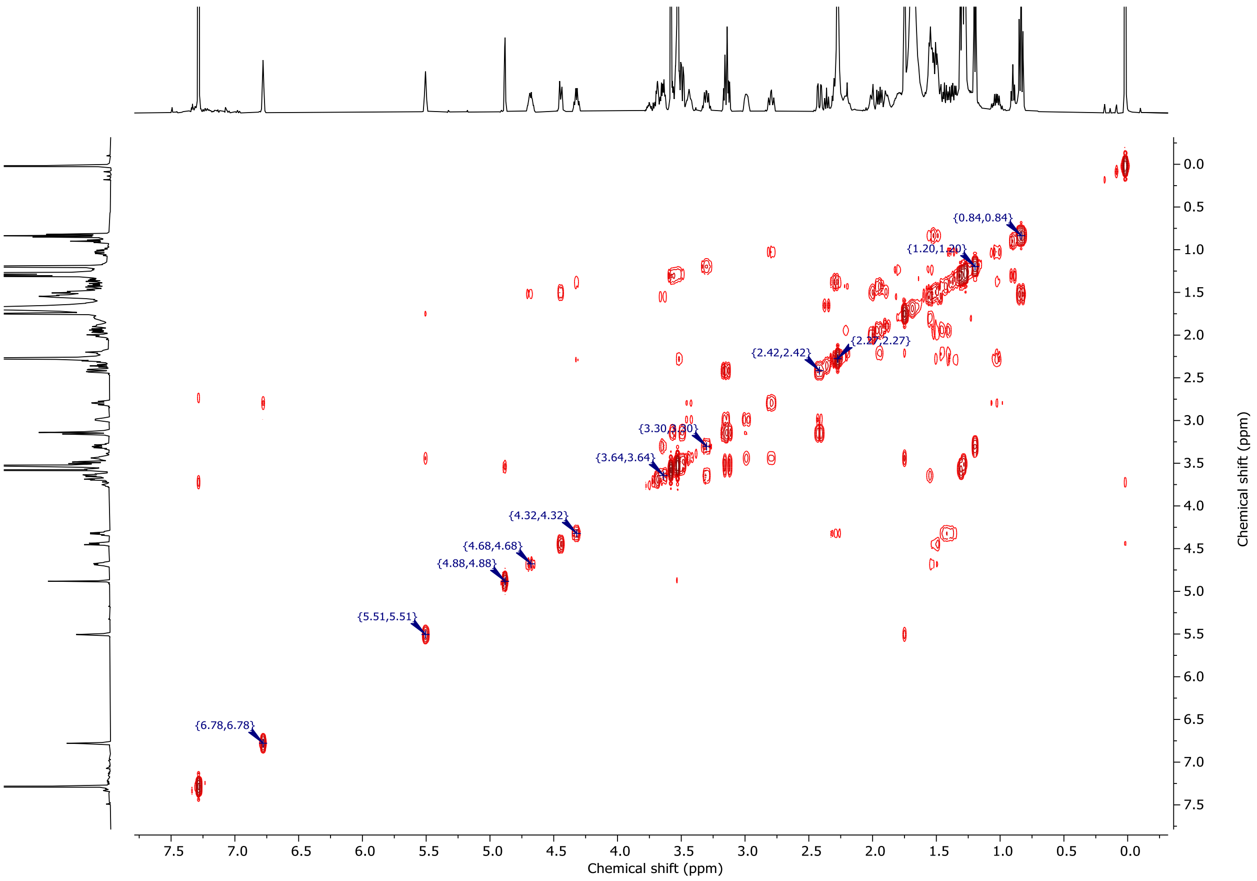

HSQC NMR spectra of Spinosyn D showing the correlations between hydrogen and carbon atoms

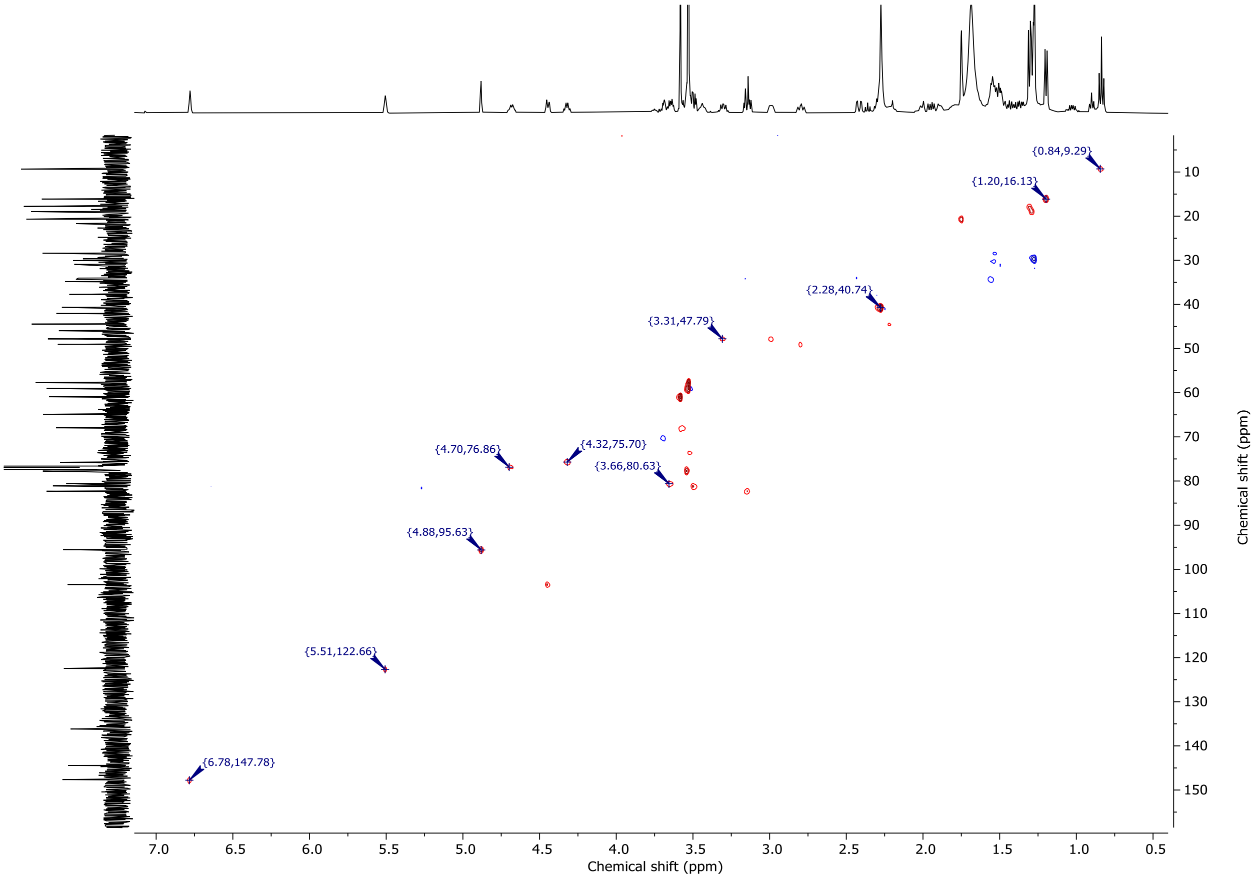

HMBC NMR spectra of Spinosyn D showing the correlations between hydrogen and carbon atoms

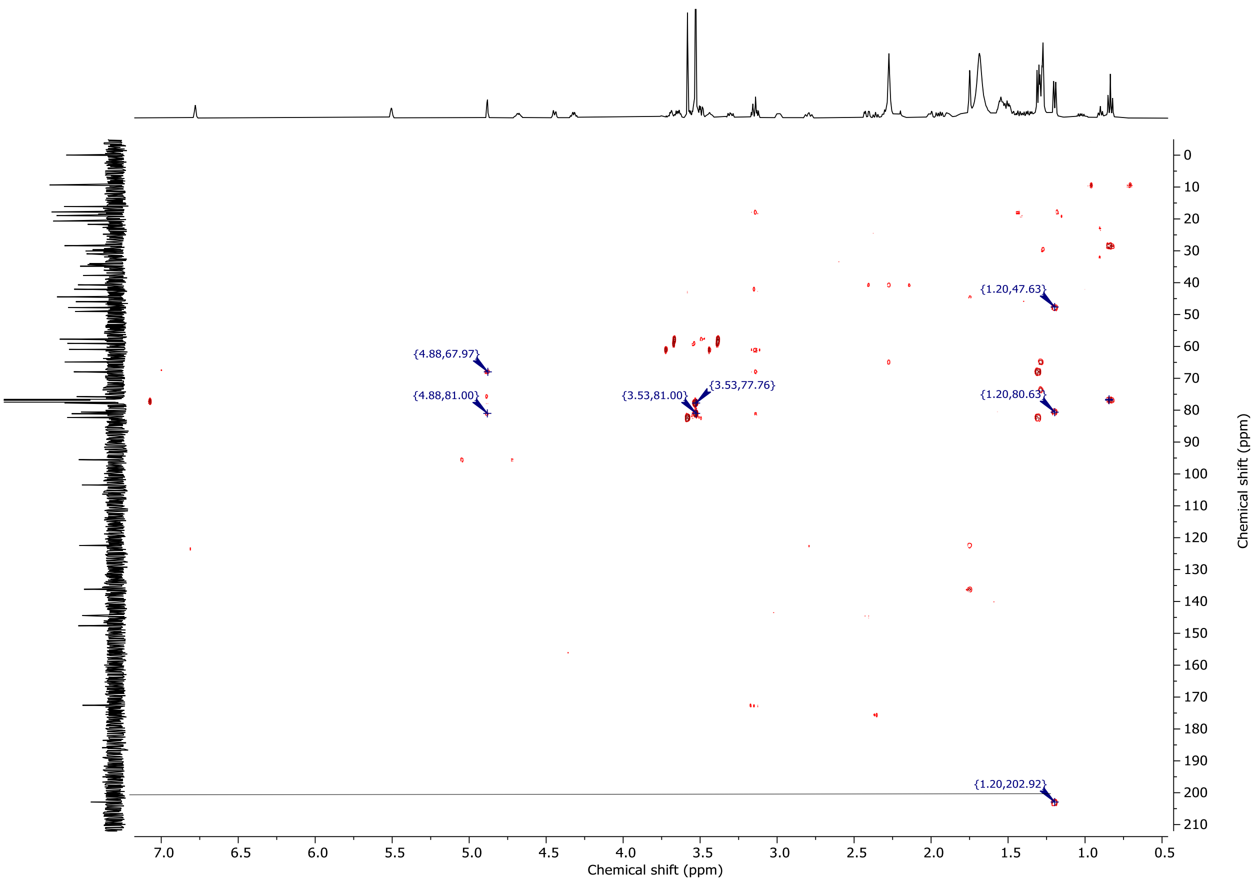

Combined ^{1}H NMR spectra of Spinosad. The first spectrum (green) represents the mixture of Spinosyns, the second spectrum (red) shows Spinosyn A, and the third spectrum (blue) displays Spinosyn D

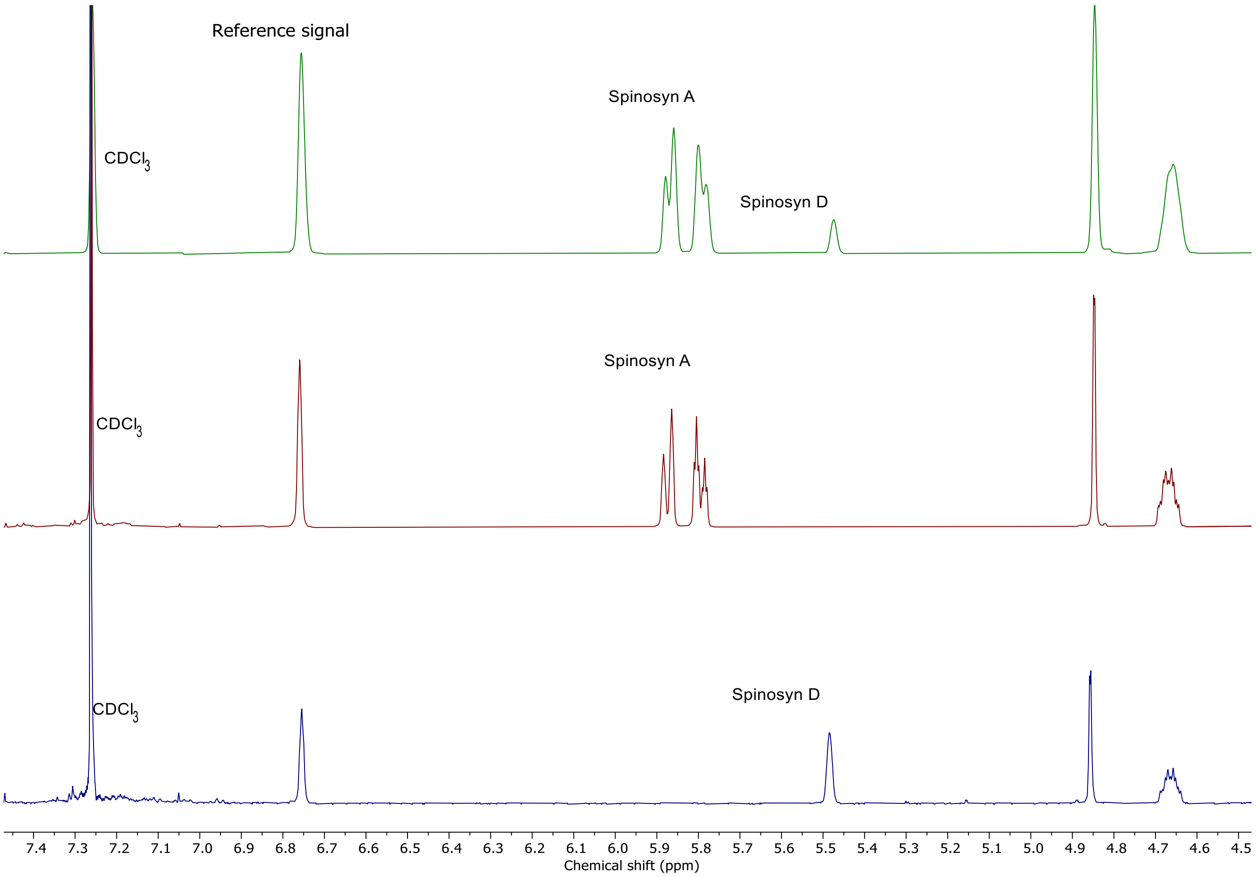

Internal standards

TMS/$CDCl_3$

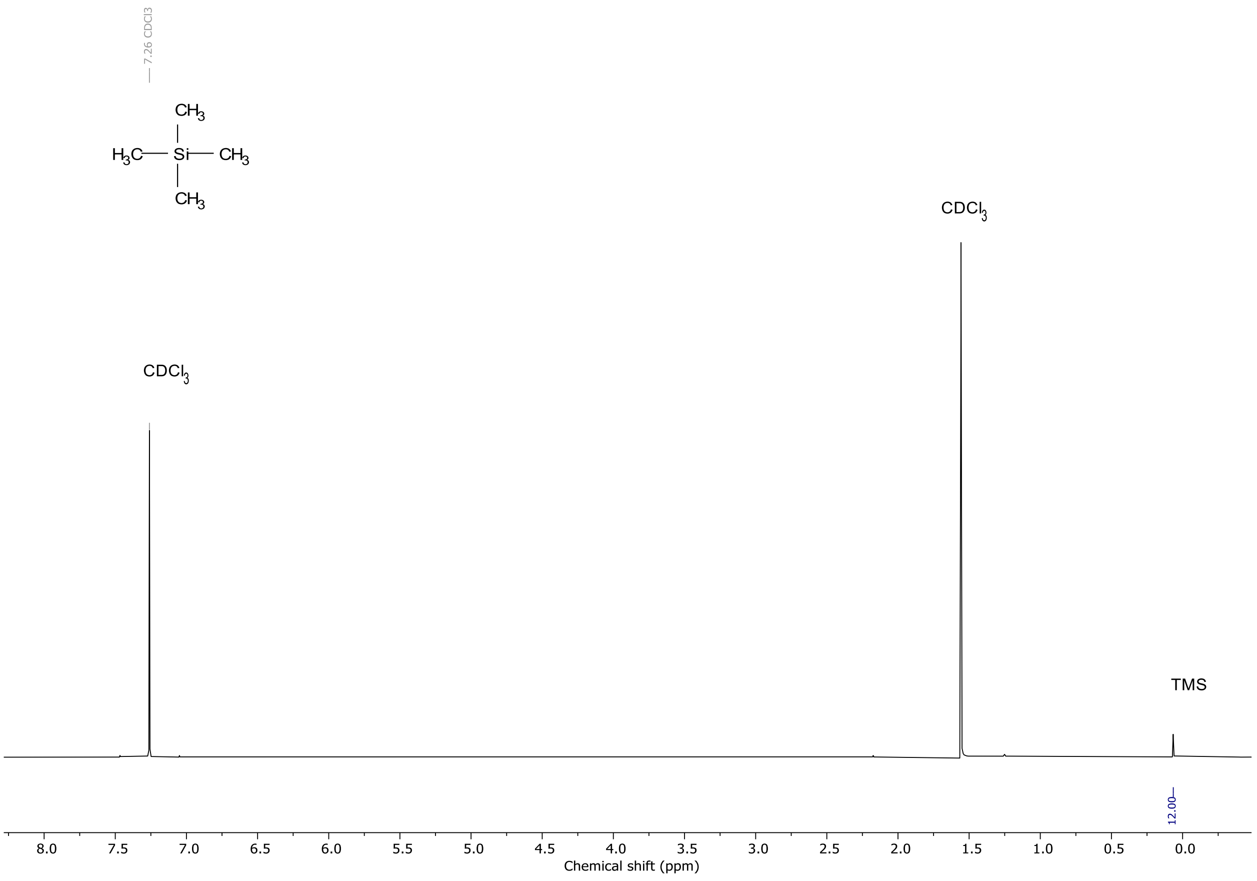

BA/$CDCl_3$

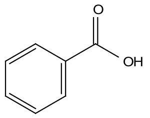

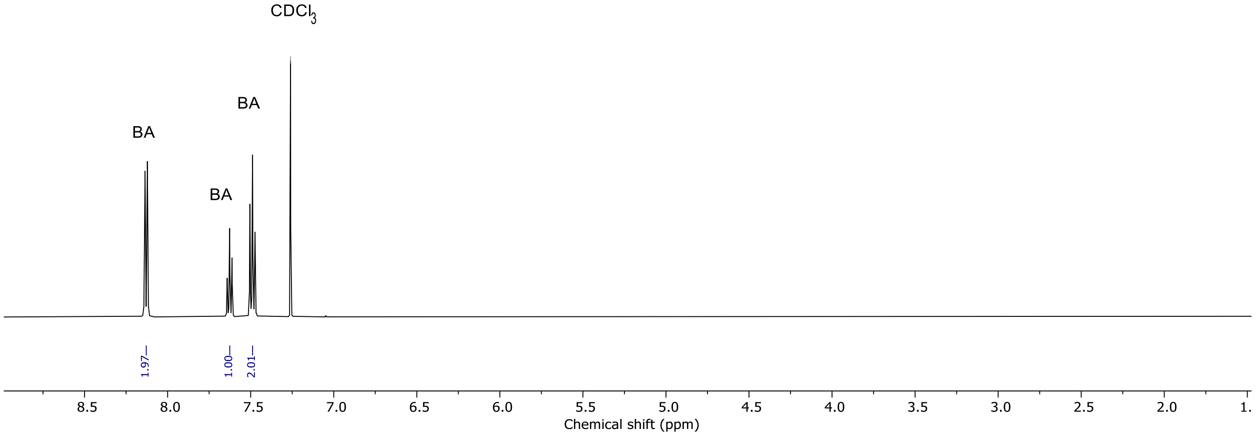
7.26 CDCl3
CDCl3
BA
BA
BA
1.97
1.00
2.01
8.5
8.0
7.5
7.0
6.5
6.0
5.5
5.0
4.5
4.0
3.5
3.0
2.5
2.0
Chemical shift (ppm)

MTC/$CDCl_3$

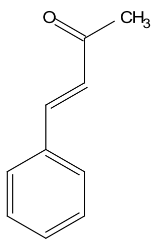

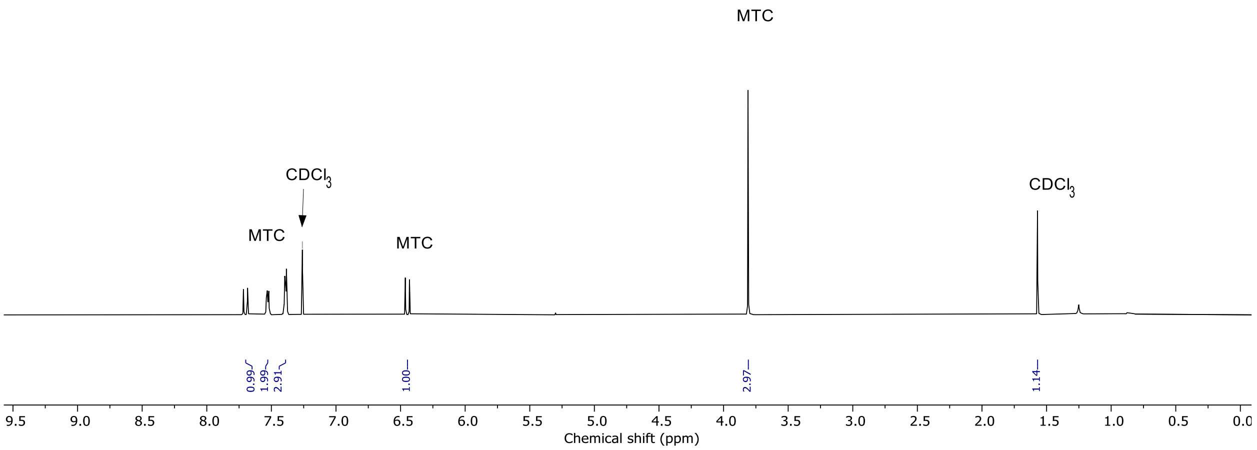

7.26 CDCl3
MTC
CDCl3
MTC
MTC
CDCl3
0.99
1.99
2.91
1.00
2.97
1.14
9.5
9.0
8.5
8.0
7.5
7.0
6.5
6.0
5.5
5.0
4.5
4.0
3.5
3.0
2.5
2.0
1.5
1.0
0.5
0.0
Chemical shift (ppm)

L-menthol/$CDCl_3$

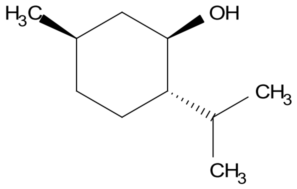

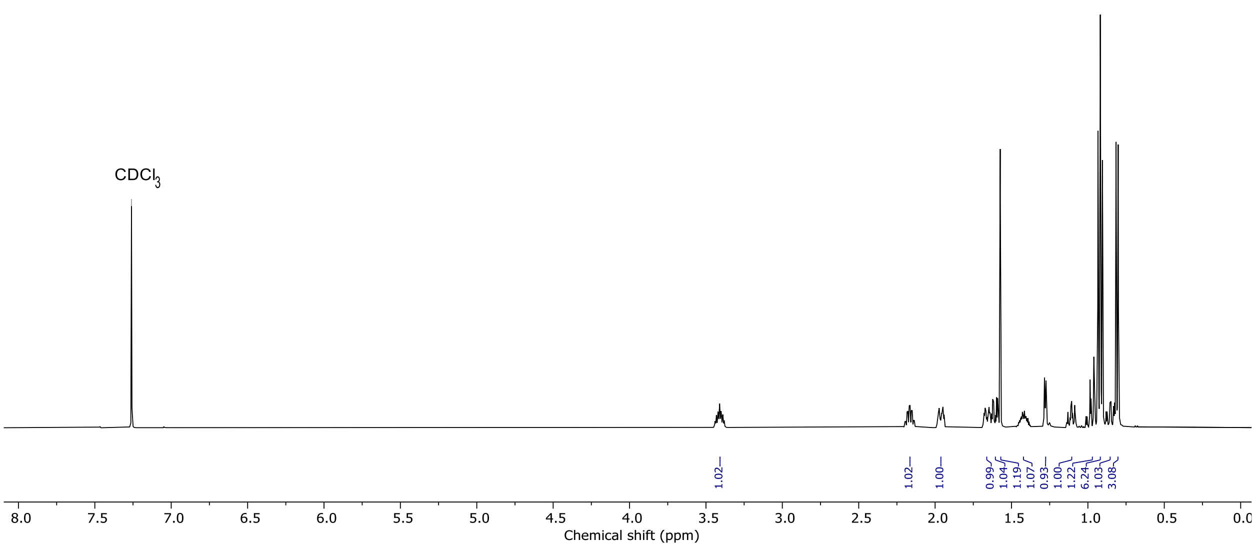

Cyclohexane/$CDCl_3$

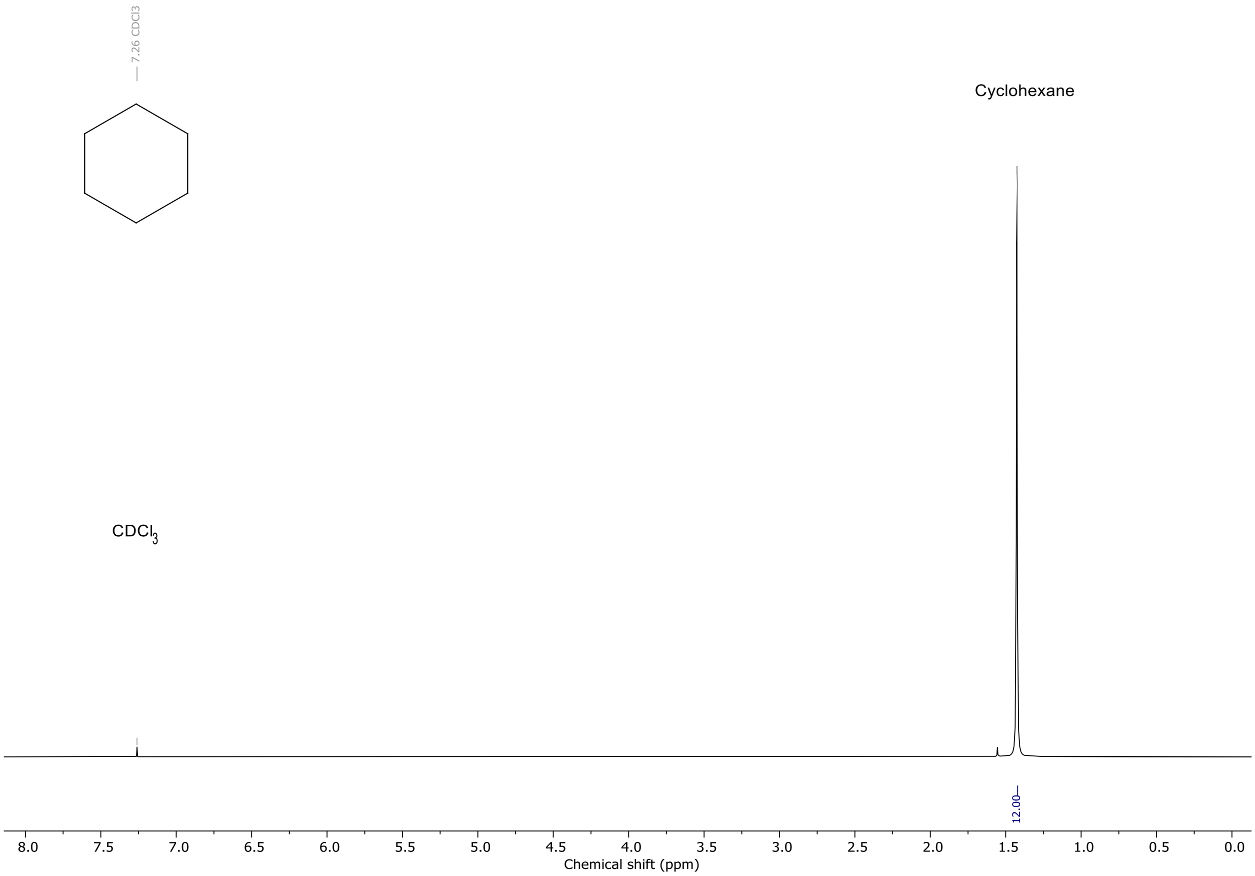

TMB/$CDCl_3$

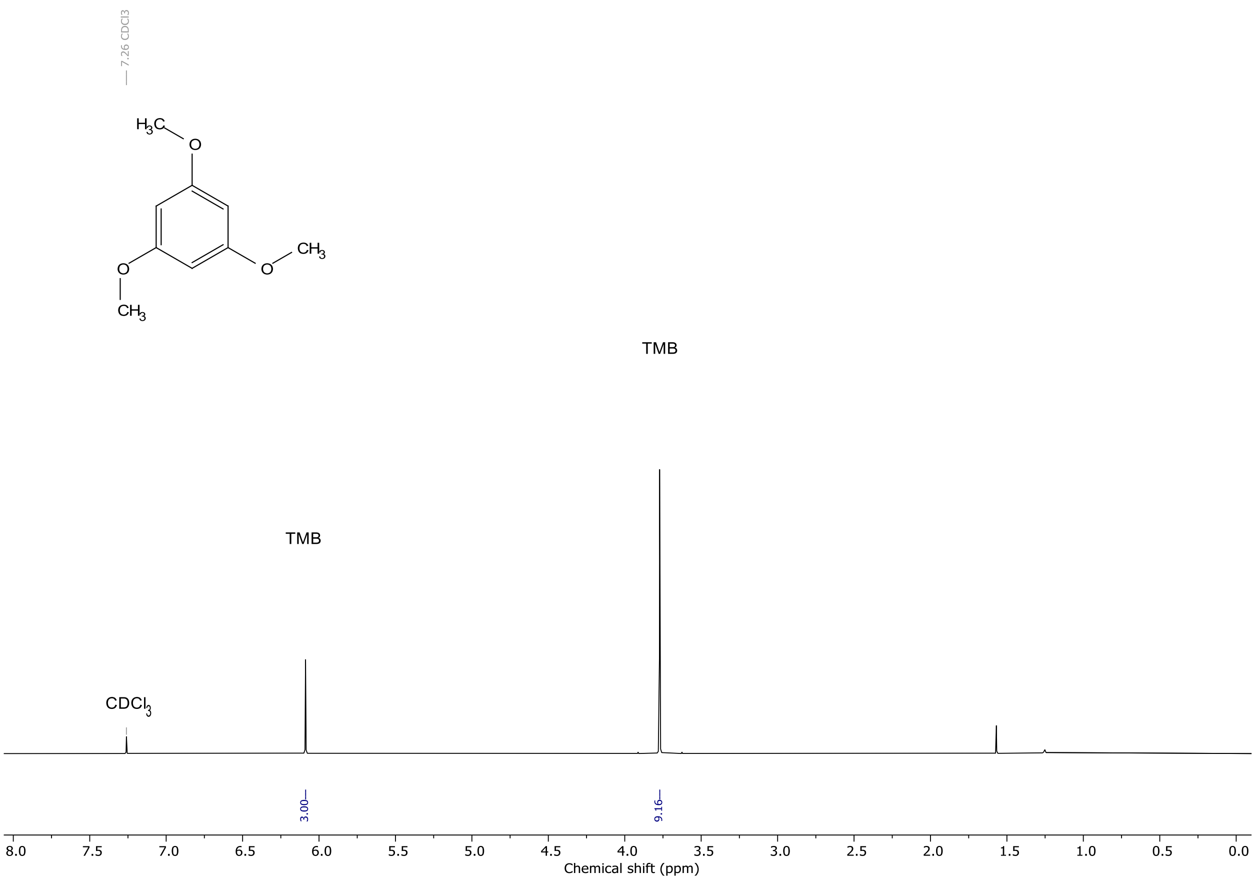

7.26 CDCl3
H3C
O
CH3
O
O
CH3
TMB
TMB
CDCl3
3.00
9.16
8.0 7.5 7.0 6.5 6.0 5.5 5.0 4.5 4.0 3.5 3.0 2.5 2.0 1.5 1.0 0.5 0.0
Chemical shift (ppm)

Quantification tests for internal standards

Internal standard BA + $CDCl_3$ + SP (1)

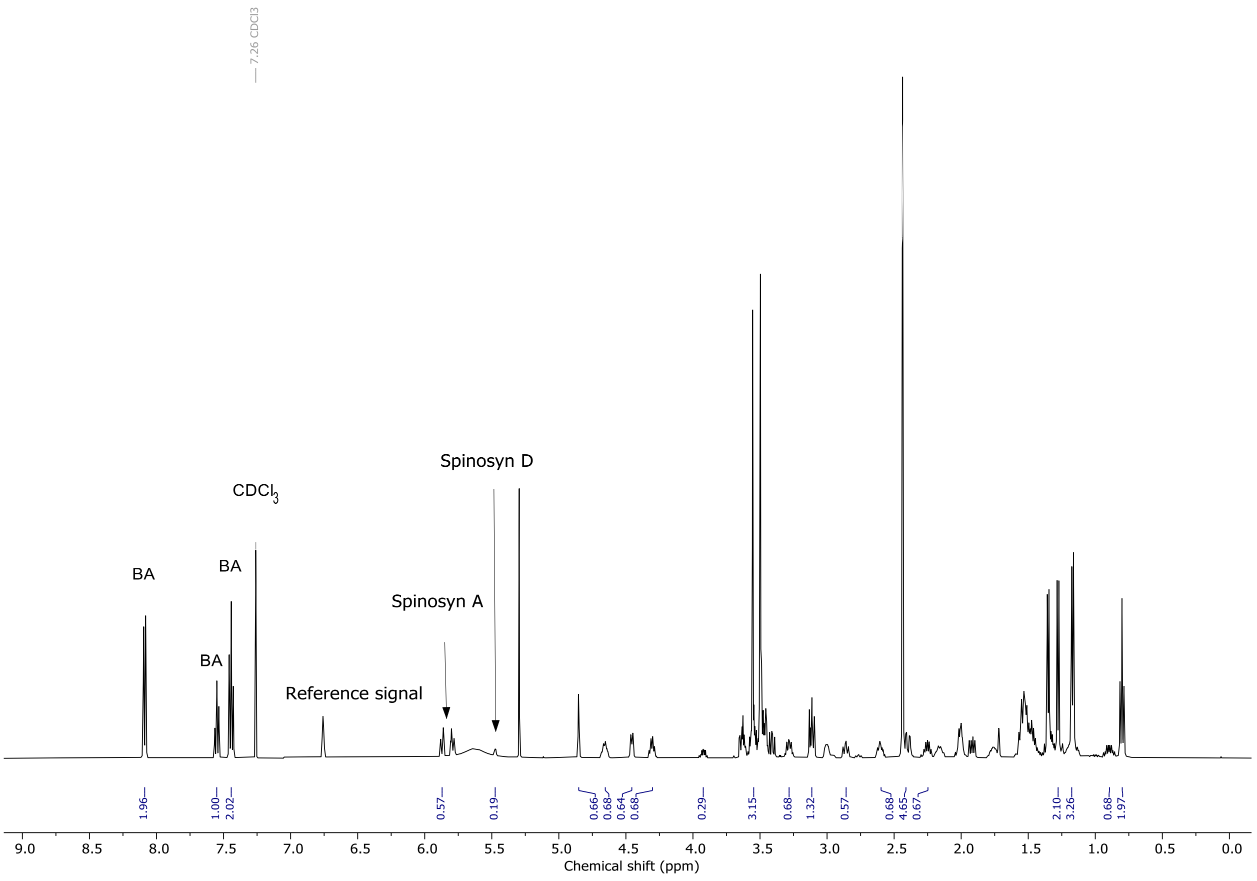

Internal standard BA + $CDCl_3$ + SP (2)

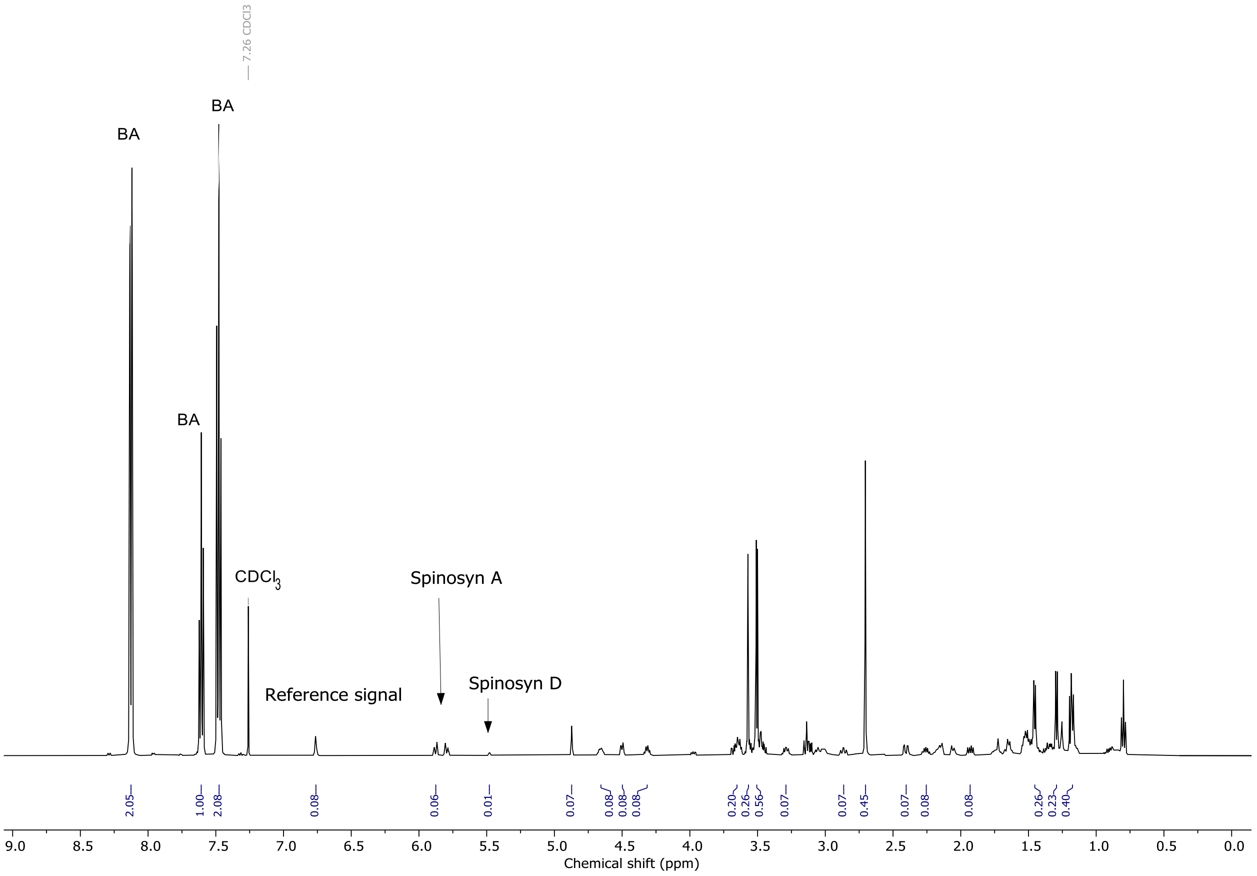

Internal standard BA + $CDCl_3$ + SP (3)

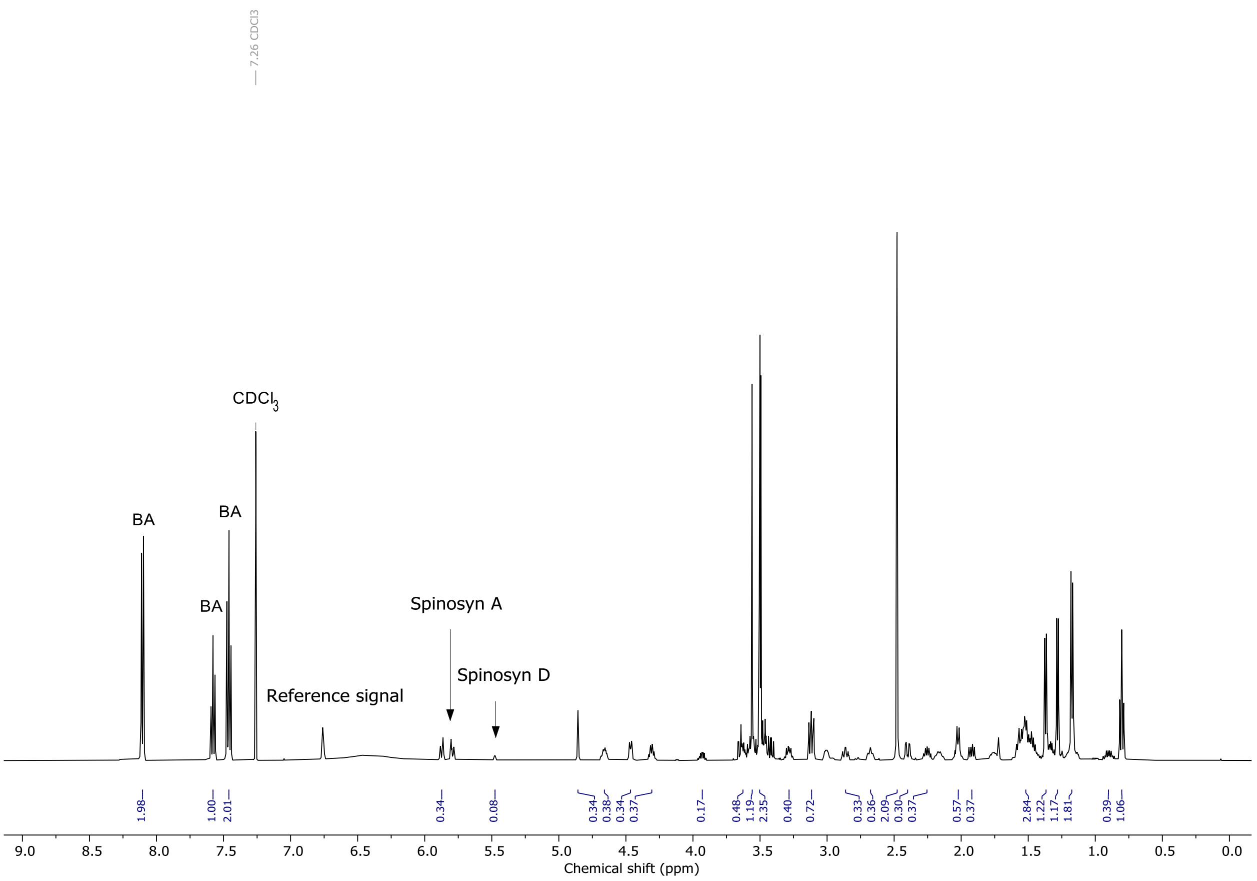

Internal standard MTC + $CDCl_3$ + SP (1)

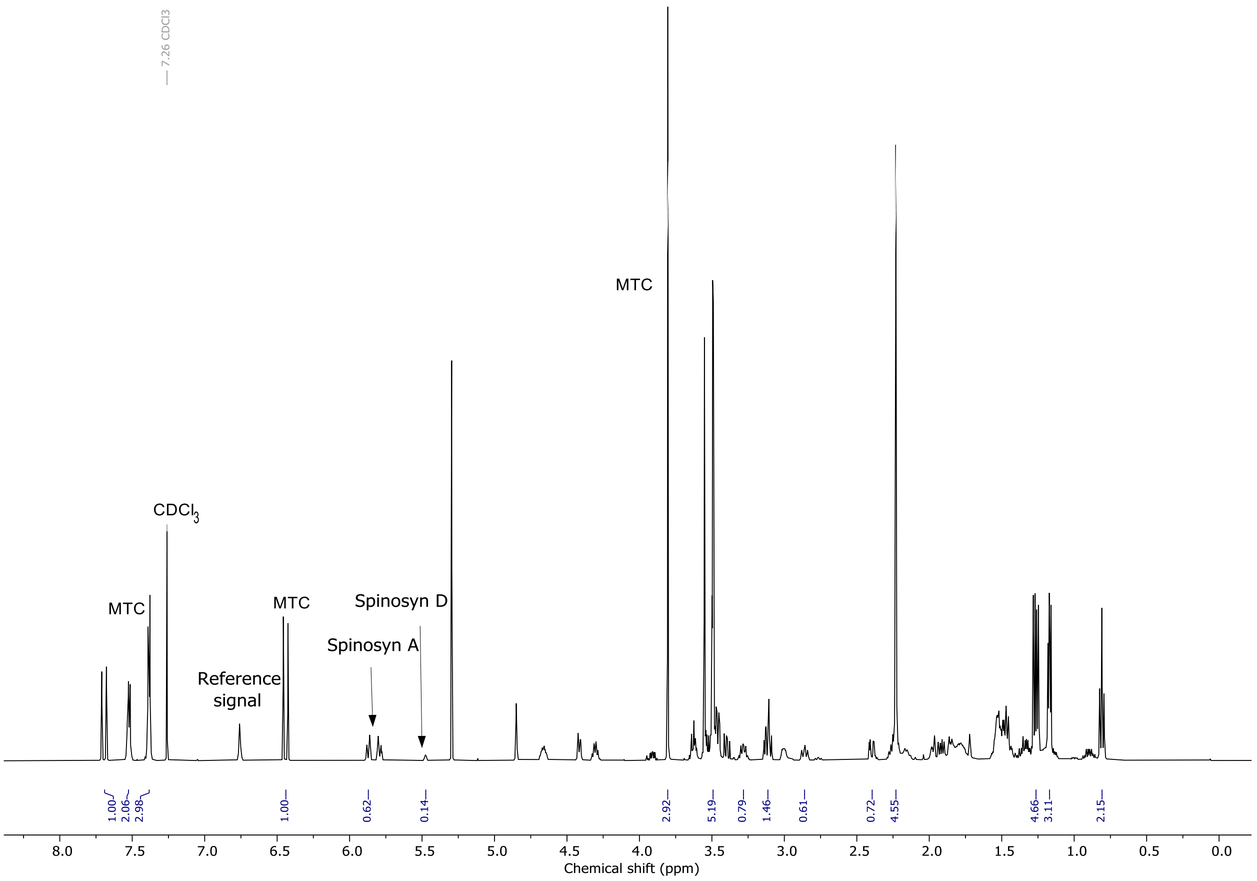

Internal standard MTC + $CDCl_3$ + SP (2)

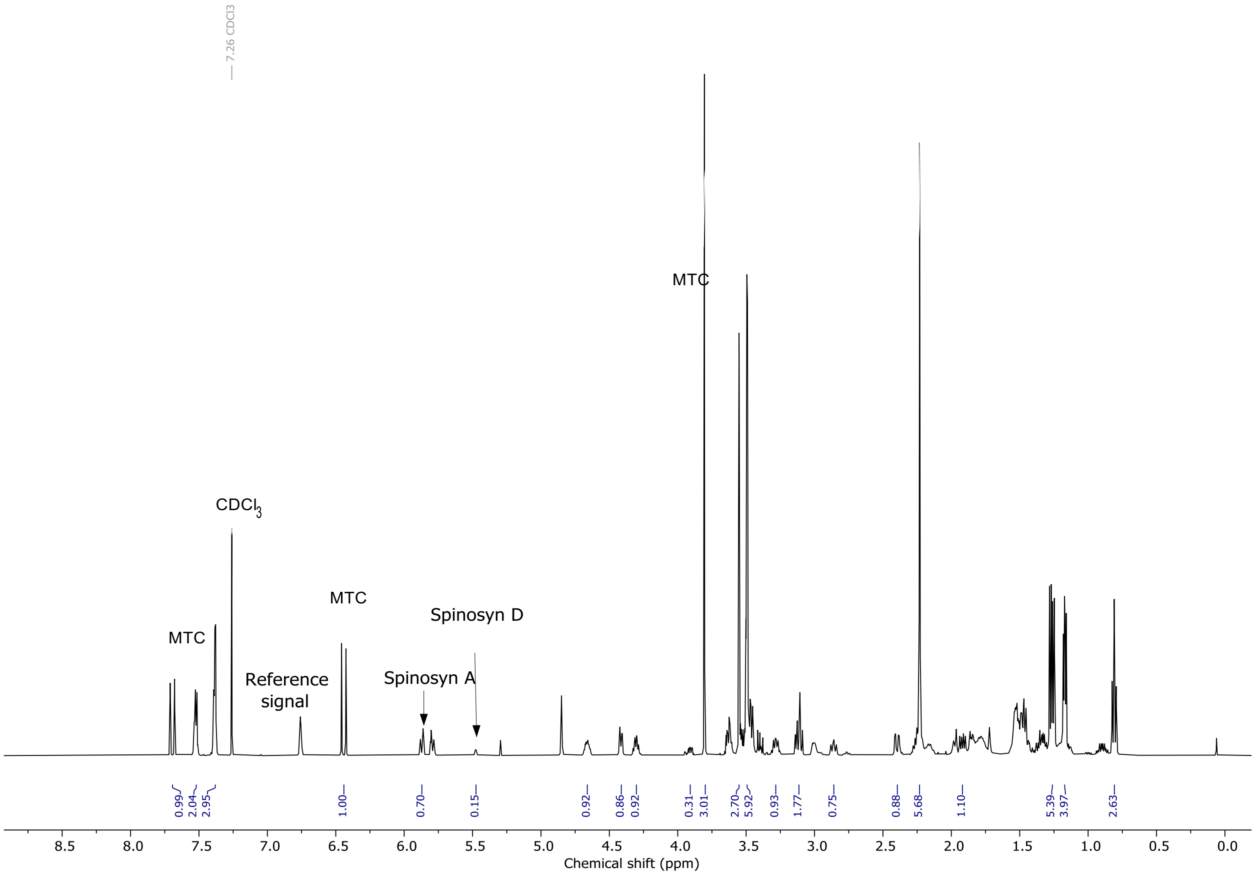

Internal standard MTC + $CDCl_3$ + SP (3)

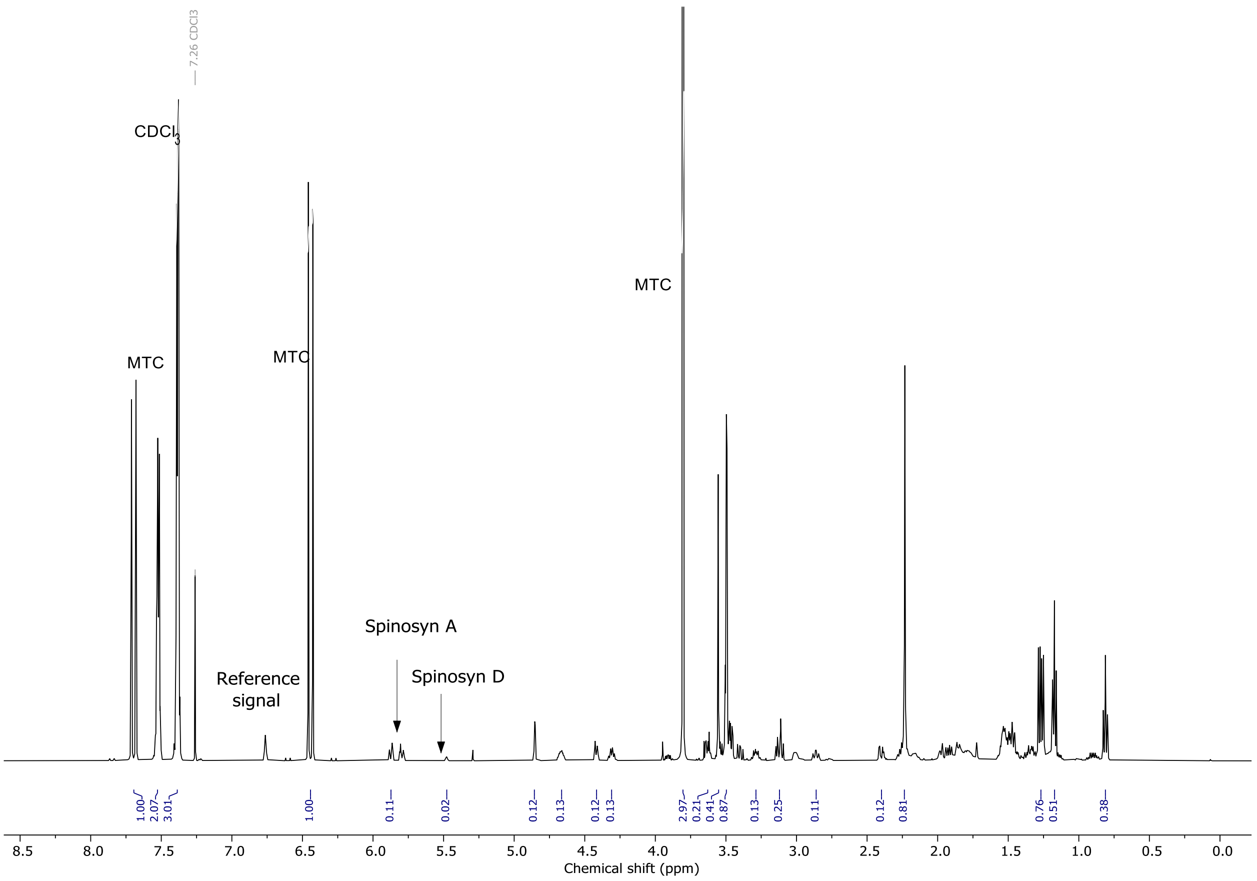

Internal standard TMB + CDCl3 + SP (1)

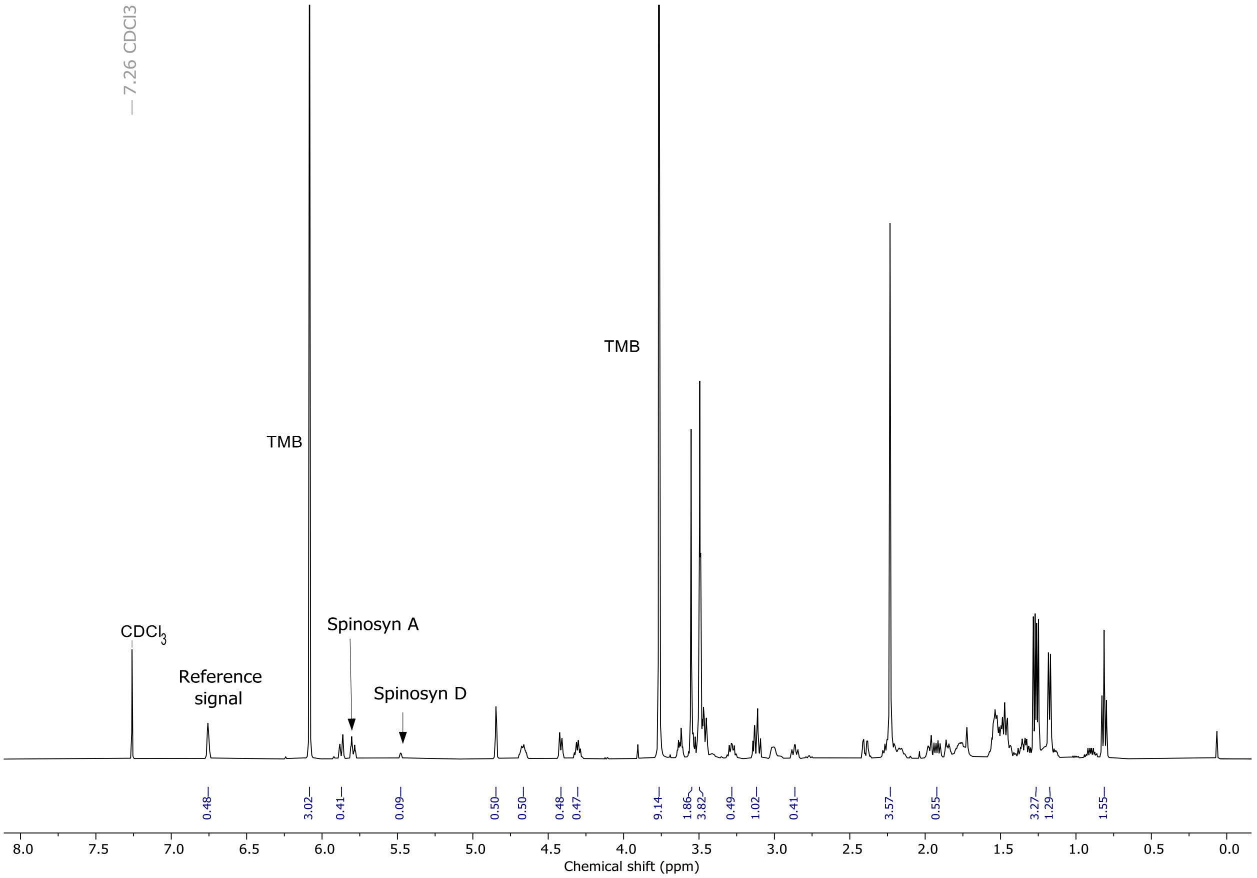

Internal standard TMB + CDCl3 + SP (2)

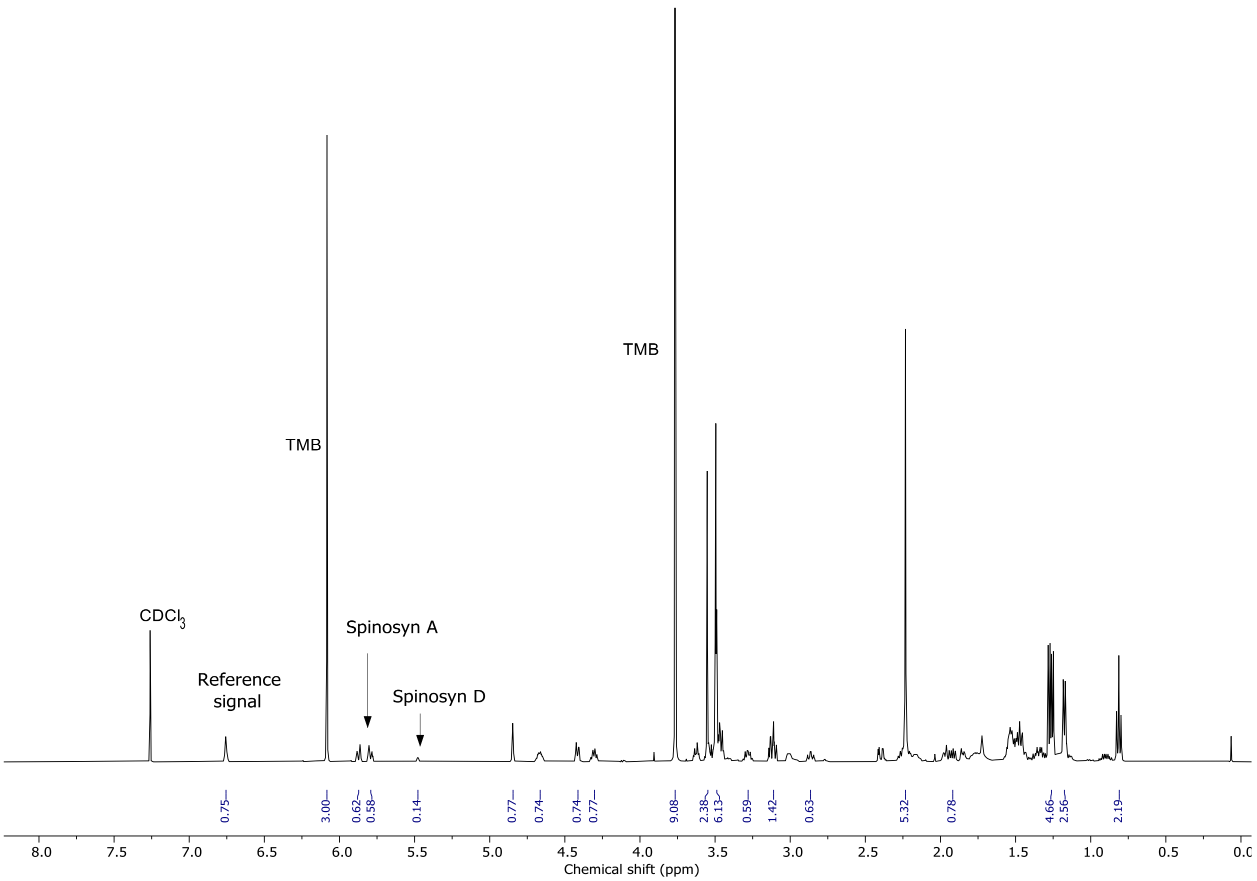

Internal standard TMB + CDCl3 + SP (3)

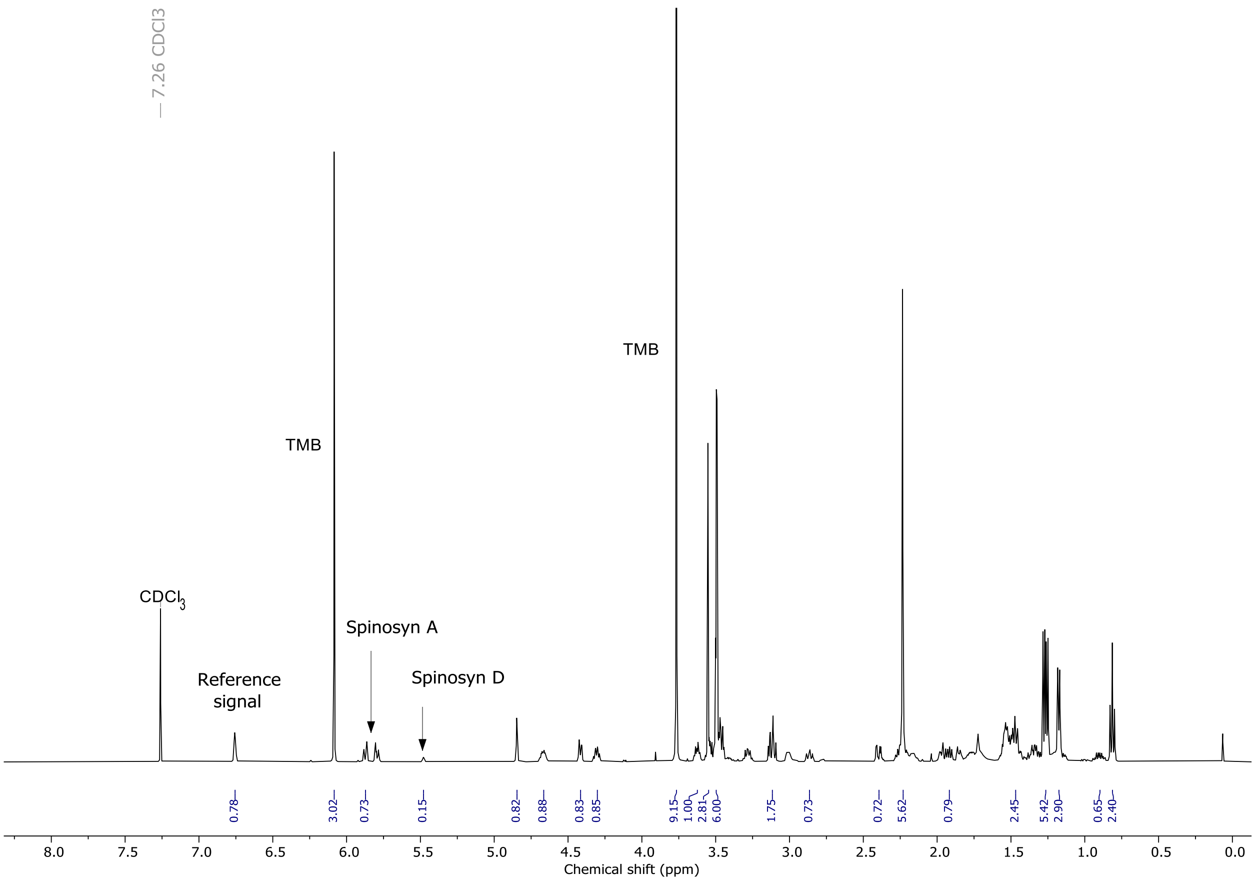

Specificity

^{1}H NMR spectra showing the signals for A) Spinosyn A, B) Spinosyn D, C) Reference ($CDCl_3$) and D) TMB

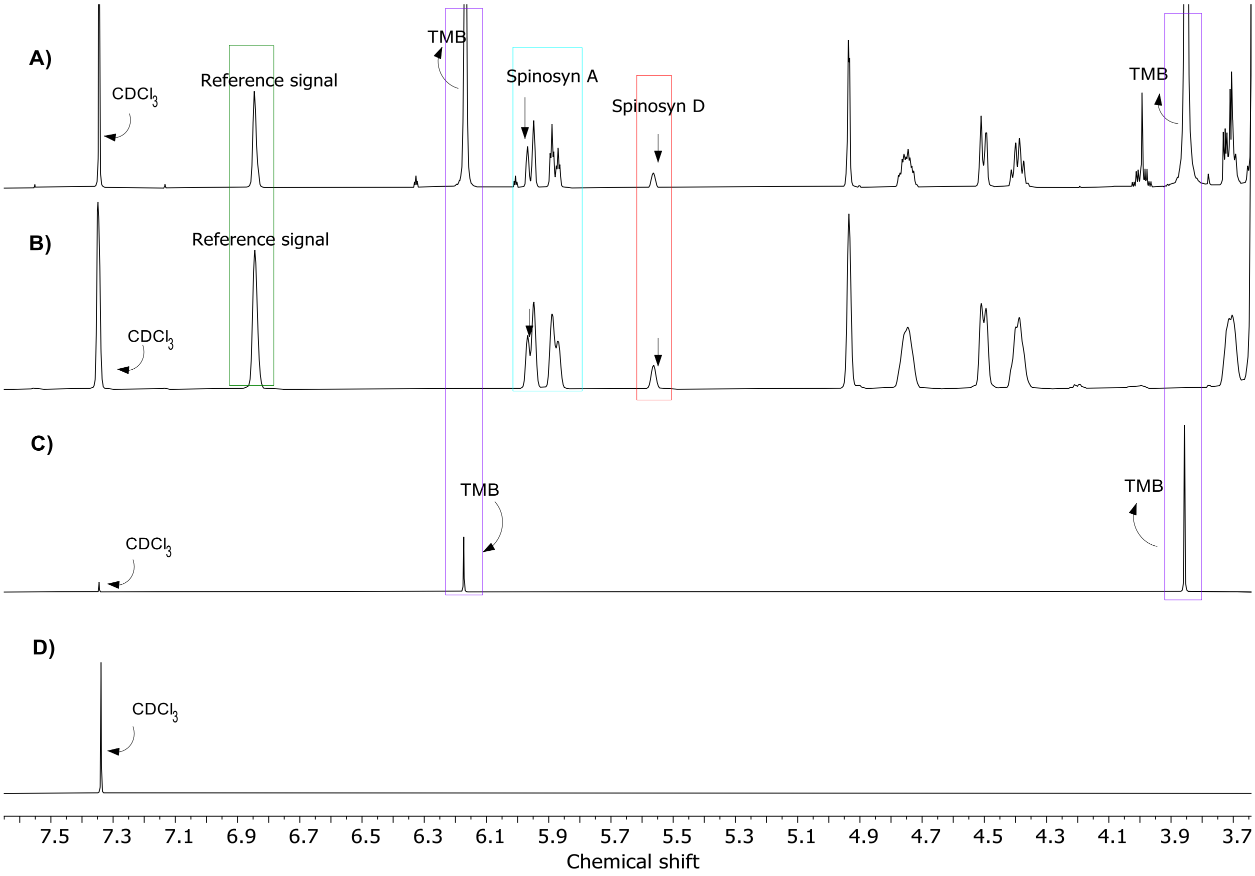

Linearity

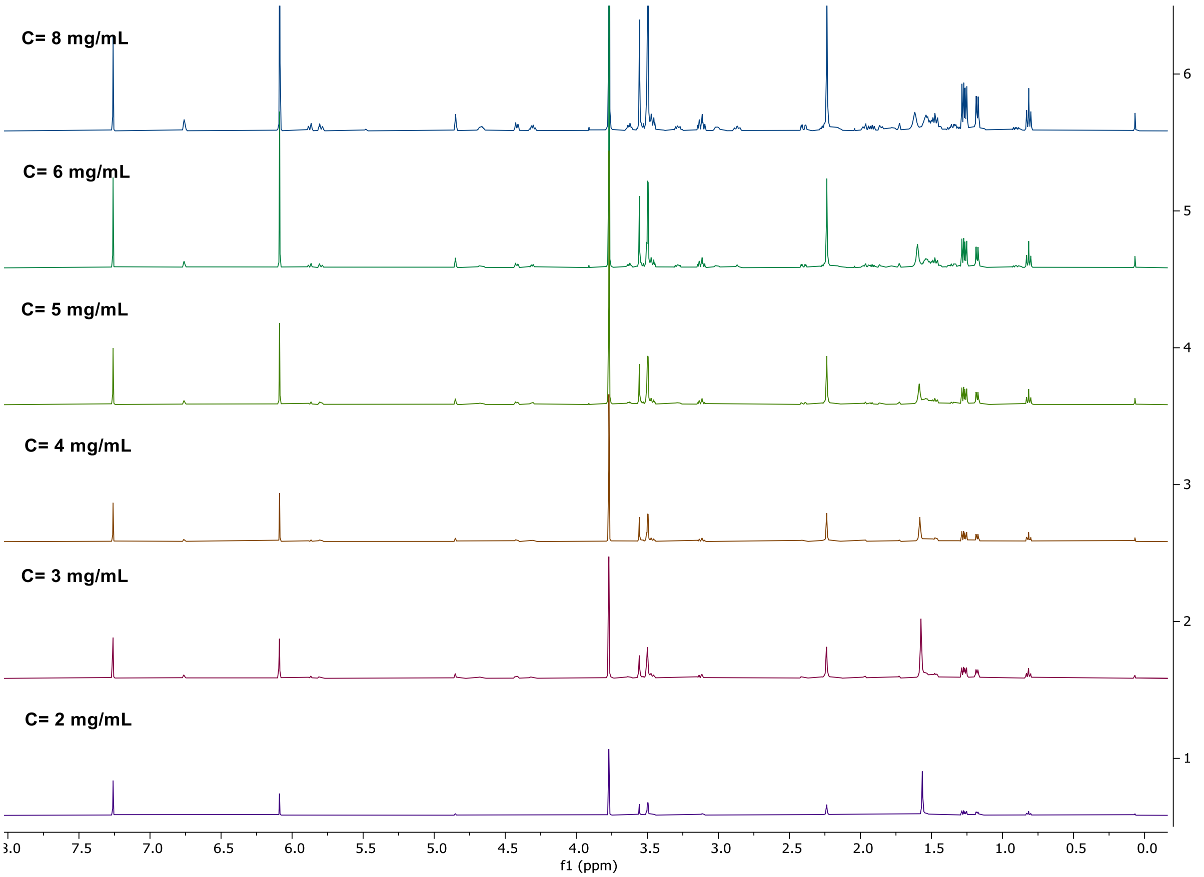

Limit of Detection and Quantification

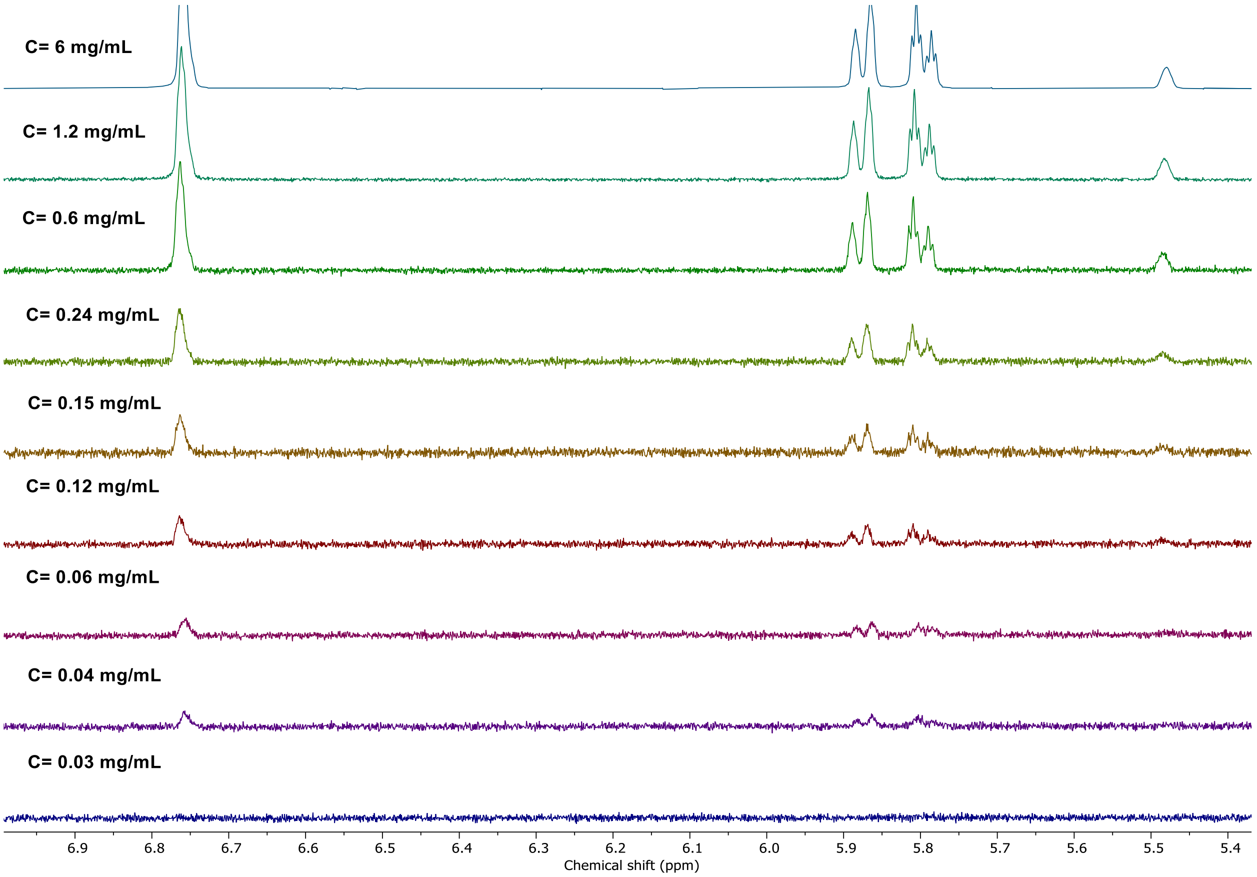

Solution Stability

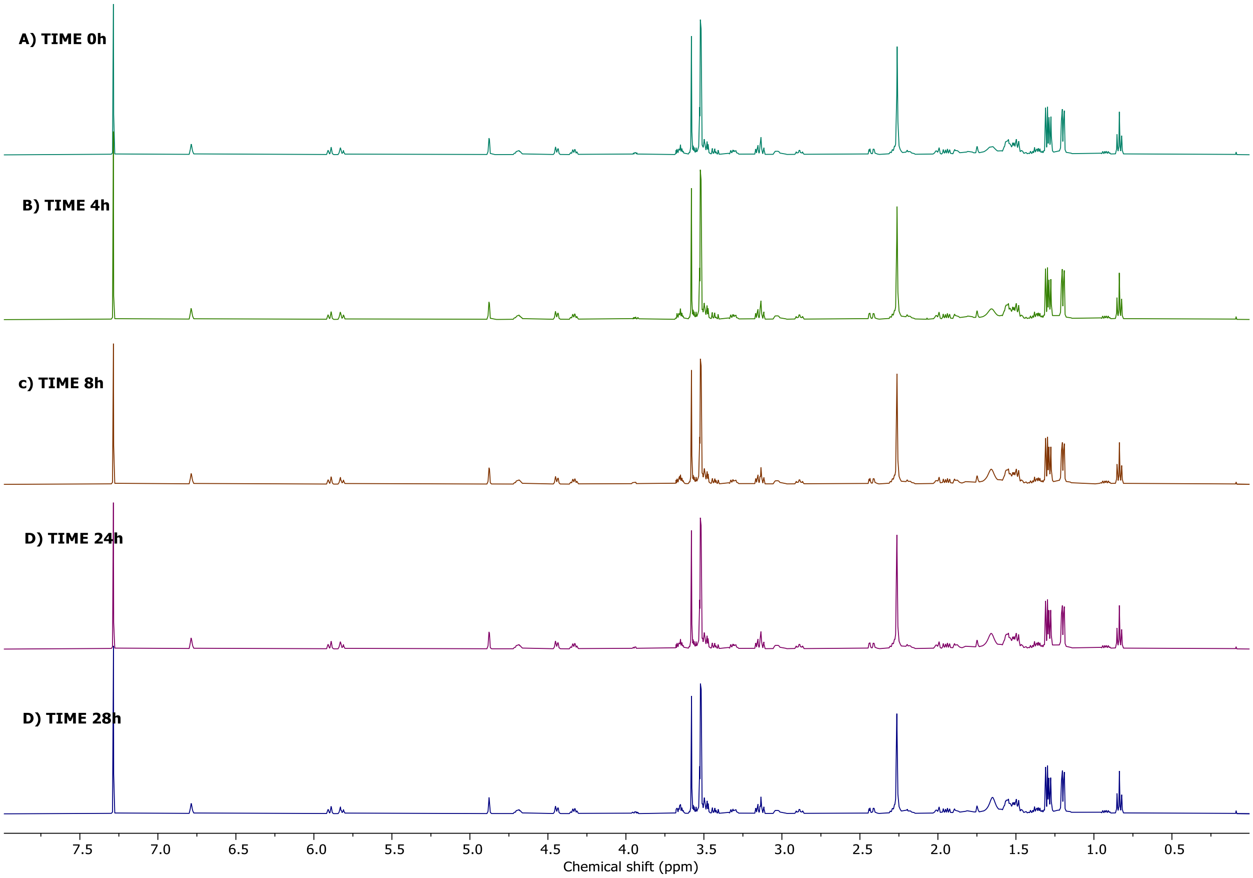

^{1}H NMR spectra of soil samples

(A) Red and (B) black soil sample

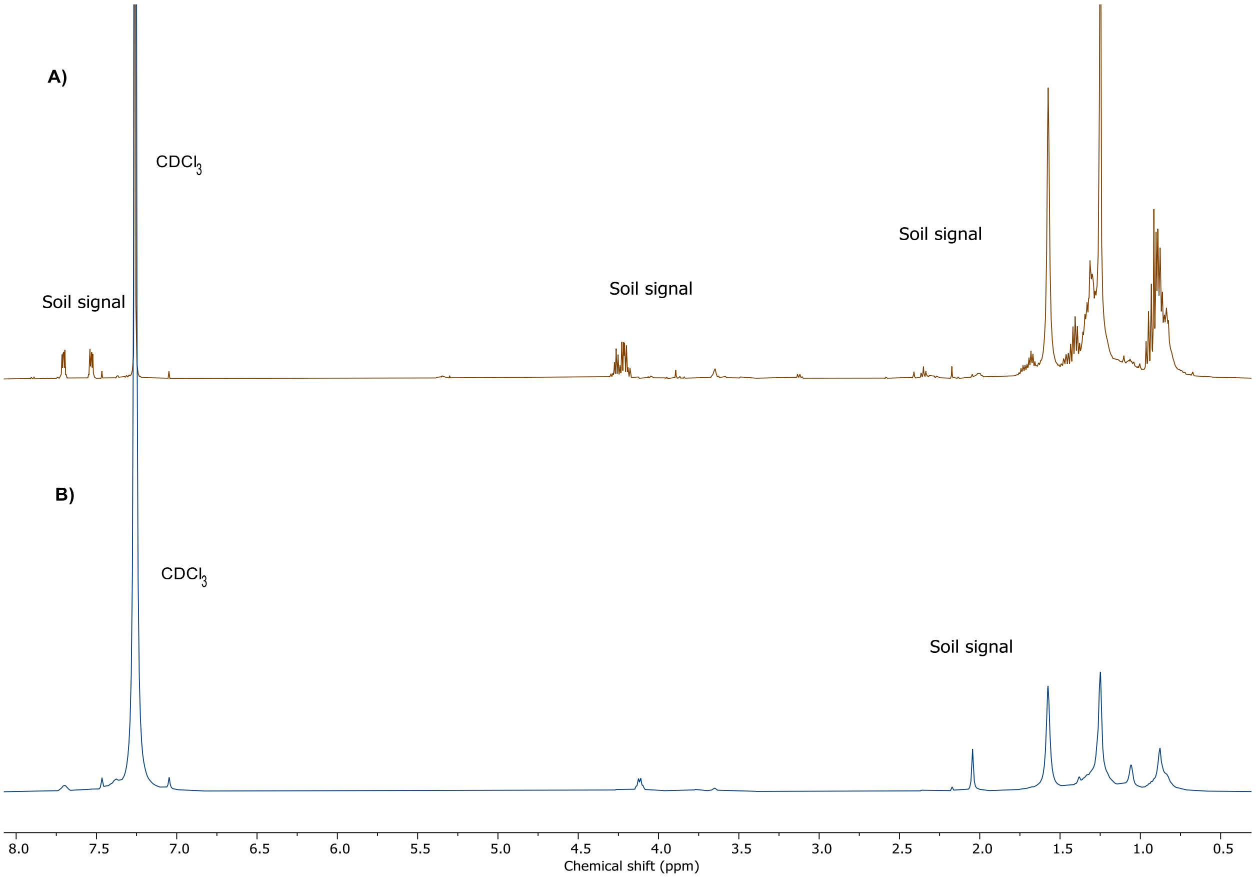

^{1}H NMR spectra showing A) Soil sample/ AcOEt, B) Soil sample/$CHCl_3$, C) Soil sample / EtOH and D) Spinosad

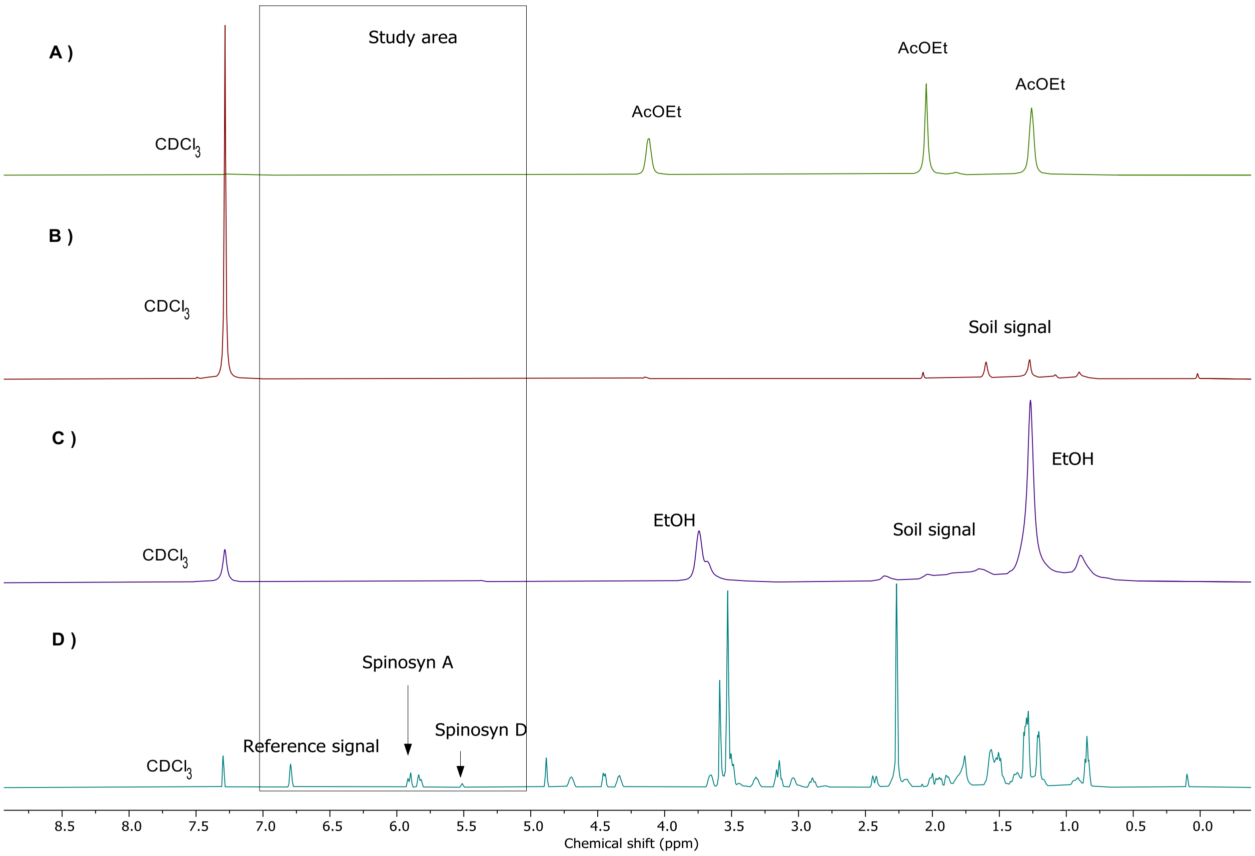

Supplement: RA-015-D5RA00356C-s001 [file RA-015-D5RA00356C-s001.pdf]
